# A sporadic Alzheimer's blood-brain barrier model for developing ultrasound-mediated delivery of Aducanumab and anti-Tau antibodies

**DOI:** 10.7150/thno.72685

**Published:** 2022-09-25

**Authors:** Joanna M. Wasielewska, Juliana C. S. Chaves, Rebecca L. Johnston, Laura A. Milton, Damián Hernández, Liyu Chen, Jae Song, Wendy Lee, Gerhard Leinenga, Rebecca M. Nisbet, Alice Pébay, Jürgen Götz, Anthony R. White, Lotta E. Oikari

**Affiliations:** 1 Cell & Molecular Biology Department, Mental Health and Neuroscience Program, QIMR Berghofer Medical Research Institute; Brisbane, QLD, Australia.; 2 Faculty of Medicine, The University of Queensland; Brisbane, QLD, Australia.; 3 Genetics & Computational Biology Department, QIMR Berghofer Medical Research Institute; Brisbane, QLD, Australia.; 4 Department of Anatomy and Physiology, The University of Melbourne; Parkville, VIC, Australia.; 5 Clem Jones Centre for Ageing Dementia Research, Queensland Brain Institute, The University of Queensland; Brisbane, QLD, Australia.; 6 The Florey Institute of Neuroscience and Mental Health; Parkville, VIC, Australia.; 7 Department of Surgery, Royal Melbourne Hospital, The University of Melbourne; Parkville, VIC, Australia.; 8 School of Biomedical Sciences, Faculty of Medicine, The University of Queensland; Brisbane, QLD, Australia.

**Keywords:** Alzheimer's disease, blood-brain barrier, focused ultrasound, drug delivery, Aduhelm

## Abstract

**Rationale:** The blood-brain barrier (BBB) is a major impediment to therapeutic intracranial drug delivery for the treatment of neurodegenerative diseases, including Alzheimer's disease (AD). Focused ultrasound applied together with microbubbles (FUS^+MB^) is a novel technique to transiently open the BBB and increase drug delivery. Evidence suggests that FUS^+MB^ is safe, however, the effects of FUS^+MB^ on human BBB cells, especially in the context of AD, remain sparsely investigated. In addition, there currently are no cell platforms to test for FUS^+MB^-mediated drug delivery.

**Methods:** Here we generated BBB cells (induced brain endothelial-like cells (iBECs) and astrocytes (iAstrocytes)) from apolipoprotein E gene allele E4 (*APOE4*, high sporadic AD risk) and allele E3 (*APOE3*, lower AD risk) carrying patient-derived induced pluripotent stem cells (iPSCs). We established mono- and co-culture models of human sporadic AD and control BBB cells to investigate the effects of FUS^+MB^ on BBB cell phenotype and to screen for the delivery of two potentially therapeutic AD antibodies, an Aducanumab-analogue (Aduhelm^TM^; anti-amyloid-β) and a novel anti-Tau antibody, RNF5. We then developed a novel hydrogel-based 2.5D BBB model as a step towards a more physiologically relevant FUS^+MB^ drug delivery platform.

**Results:** When compared to untreated cells, the delivery of Aducanumab-analogue and RNF5 was significantly increased (up to 1.73 fold), across the Transwell-based BBB models following FUS^+MB^ treatment. Our results also demonstrated the safety of FUS^+MB^ indicated by minimal changes in iBEC transcriptome as well as little or no changes in iBEC or iAstrocyte viability and inflammatory responses within the first 24 h post FUS^+MB^. Furthermore, we demonstrated successful iBEC barrier formation in our novel 2.5D hydrogel-based BBB model with significantly increased delivery (1.4 fold) of Aducanumab-analogue following FUS^+MB^.

**Conclusion:** Our results demonstrate a robust and reproducible approach to utilize patient cells for FUS^+MB^-mediated drug delivery screening *in vitro*. With such a cell platform for FUS^+MB^ research previously not reported, it has the potential to identify novel FUS^+MB^-deliverable drugs as well as screen for cell- and patient-specific effects of FUS^+MB^, accelerating the use of FUS^+MB^ as a therapeutic modality in AD.

## Introduction

Alzheimer's disease (AD) is the most prevalent cause of dementia characterized by progressive and irreversible cognitive decline, including loss of short- and long-term memory 1. The majority of AD cases (>95%) are described as sporadic with late disease onset (over 65 years of age) and a genetic etiology that is not fully understood. However, several genetic risk factors have been identified for sporadic AD, including the E4 allele of the apolipoprotein E (*APOE*) gene 2, 3. Homozygote carriers of the *APOE4* allele have a 15-fold increased risk of developing AD compared to the most common isoform *APOE3* when homozygous 2, 3. The most commonly described pathological hallmarks of AD include the accumulation of amyloid-β plaques (Aβ) and phosphorylated-Tau (p-Tau) containing neurofibrillary tangles (NFTs) 1, 4, 5. However, emerging evidence indicates that changes in the brain vasculature are also associated with AD 6.

The blood-brain barrier (BBB) is a selectively permeable barrier at the blood-brain interface, formed by brain endothelial cells (BECs) and supporting cells, including astrocytes and pericytes, and its role is to maintain a highly controlled internal brain milieu 7. High integrity of the BEC monolayer is essential for barrier function of the BBB and is achieved by the high expression of tight junction (TJ) and adherens junction (AJ) proteins between adjacent BECs, including occludin, claudin-5, zona occludens-1 (ZO-1) and vascular endothelial (VE)-cadherin, respectively 8. Synergistically, TJ and AJ proteins ensure a high trans-endothelial electrical resistance (TEER) of the BBB and limit paracellular permeability through the BBB into the brain 7, 9, 10. Recently, an association between the *APOE4* isoform and BBB breakdown has been reported 11. It has been suggested that BBB dysfunction in *APOE4* carriers occurs before cognitive decline and is independent of Aβ and p-Tau accumulation, and thus an additional driving force of cognitive impairment 11. Studies in transgenic mice further support a role for *APOE4* in BBB dysfunction, reflected by early BBB breakdown and cerebral microhemorrhages, which lead to secondary neurodegeneration 8, 11-13.

The BBB is not only altered in AD, but it also poses a major physical obstacle for drug delivery into the brain. The BBB actively restricts the entry of over 98% of small molecule drugs and up to almost 100% of large therapeutic antibodies and growth factors into the brain, presenting a key hurdle in successful AD drug discovery 14. Furthermore, the altered brain milieu caused by BBB disruption in AD likely further hinders controlled drug delivery and metabolization 6, posing an urgent need for adequate AD *in vitro* and preclinical models to understand AD-specific changes in the BBB.

With a concerning 99.6% failure rate of clinical trials targeting AD, a paradigm-shift may originate from the development of accurate human-like pre-clinical models of drug delivery that improve the penetration of therapeutics through the BBB 15. Correspondingly, a recent study by Ohshima *et al.* compared the permeability of several drugs of known pharmacokinetics in various *in vitro* BBB models, showing that human induced pluripotent stem cell (hiPSC)-derived BBB models correlated most closely with human *in vivo* BBB drug permeability compared to rat BBB and Caco-2 models 16. This suggests that patient-derived iPSC-based BBB models provide a clinically relevant platform for permeability studies in AD drug discovery.

Low-intensity focused ultrasound (FUS) applied together with microbubbles (MBs) is emerging as a novel technique to transiently open the BBB to aid in drug delivery into the brain. The ultrasound wave causes intravenously injected MBs to expand and contract, and when in contact with the BBB, exert a force that results in the loosening of tight junctions leading to transient BBB opening 17. We recently demonstrated the ability to model FUS^+MB^-mediated drug delivery and Aβ clearance *in vitro* using a familial AD iPSC-derived induced brain endothelial cell-like (iBEC) model 18. Our findings reported differences in the immediate and long-term responses to FUS^+MB^ in familial AD compared to control iBECs, suggesting disease- and patient-specific effects. Importantly, the safety of ultrasound-mediated BBB opening has been demonstrated in multiple animal and several small patient studies of neurodegenerative diseases 19-25. In addition, a number of animal studies have demonstrated FUS^+MB^-mediated drug delivery *in vivo* 26-28, although drug delivery in AD patients has not been pursued to date. As a detailed understanding of the mechanisms and long-term safety of FUS^+MB^-mediated human-BBB opening and its secondary effects is still largely lacking 29, a patient cell platform to study the effects of FUS^+MB^ and identify FUS^+MB^-deliverable drugs, especially for sporadic AD, would be an important step in improving and tailoring FUS-therapies for AD patients.

Using patient-derived iPSCs is important as they can complement findings from transgenic mouse models, which can lack human disease resemblance and demonstrate structural and molecular differences to the human BBB 30-33. Thus, we developed a sporadic AD patient iPSC-derived BBB-like *in vitro* model to study the effects of FUS^+MB^ on cellular responses and drug delivery. We utilized *APOE3-* and *APOE4-* carrying iPSC-derived BBB cells and investigated molecular changes following FUS^+MB^ as well as the FUS^+MB^-mediated delivery of two therapeutic AD antibodies: an Aducanumab analogue (anti-Aβ antibody, commercially known as Aduhelm^TM^) 34, 35, and RNF5, an anti-Tau antibody, which has recently been shown to be efficient in reducing p-Tau levels in an animal model with Tau pathology 36. Here, we report the improved delivery of these antibodies in our BBB-like *in vitro* model using FUS^+MB^ and the cell-specific effects FUS^+MB^ confers in an *APOE3*/*APOE4* context.

## Results

### Human iPSC-derived *APOE4* iBECs demonstrate key phenotypical differences compared to *APOE3* iBECs

**Figure 1:**
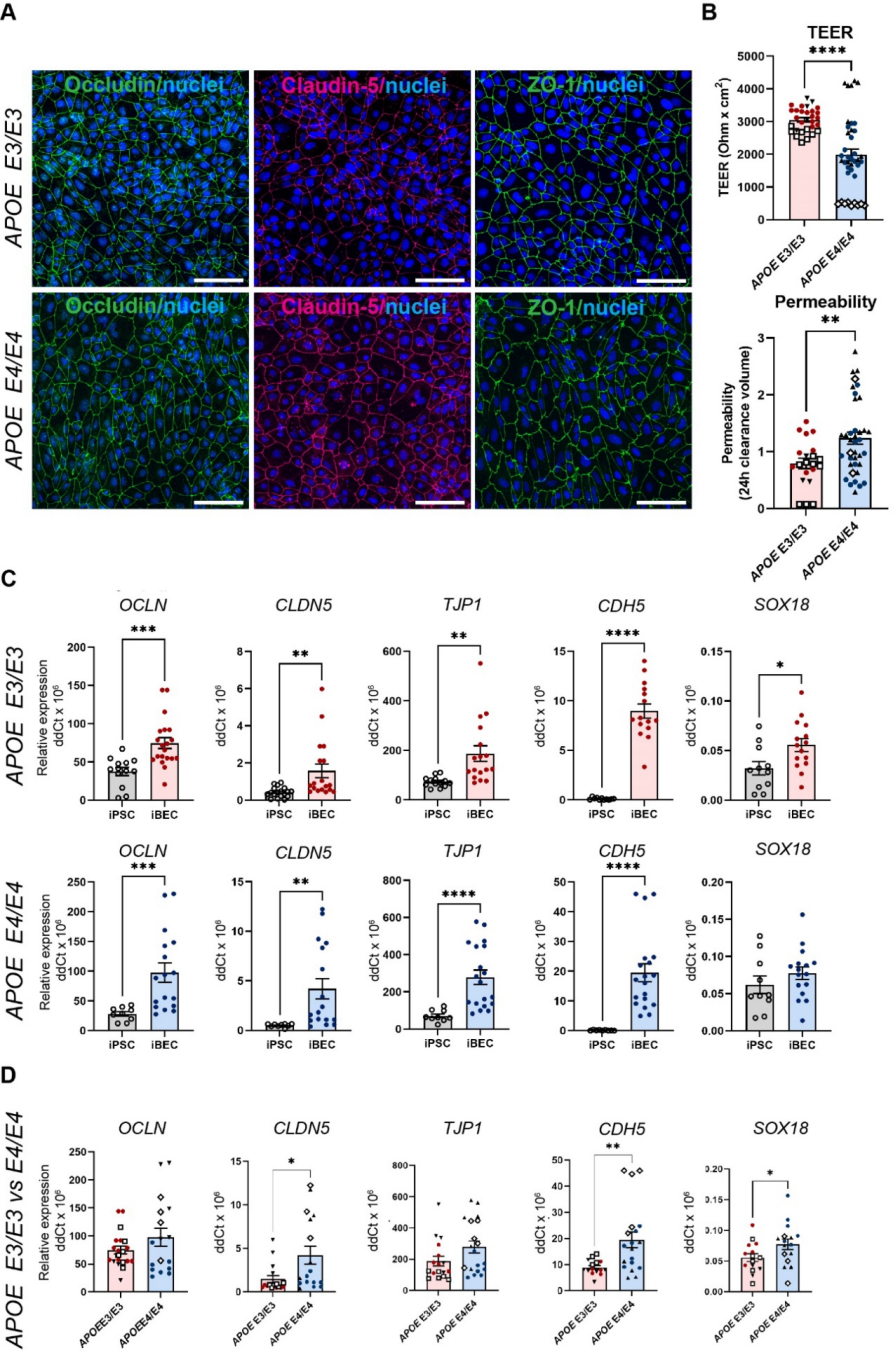
** Characterization of brain endothelial-like cells (iBECs) from *APOE3* and *APOE4*-carrying human iPSCs.** (**A**) Representative immunofluorescence images of Occludin (green), Claudin-5 (magenta) and ZO-1 (green) in *APOE3* and *APOE4* iPSC-derived iBECs (scale bar = 100 µm, nuclei stained using Hoechst). (**B**) Trans-endothelial electrical resistance (TEER, Ohm x cm^2^) and passive 5 kDa dextran permeability (24 h clearance volume) in *APOE3* and *APOE4* iBECs (separate cell lines shown as different symbols). (**C**) Relative gene expression of blood-brain barrier (BBB) and endothelial cell markers in *APOE3* iPSCs vs iBECs (top row) and *APOE4* iPSCs vs iBECs (bottom row). (**D**) Relative gene expression of BBB and endothelial cell markers in *APOE3* and *APOE4* iBECs (separate cell lines shown as different symbols). N = 3 biological replicates and a minimum of n = 3 independent replicates per line. * *P <* 0.05, ** *P <* 0.01, **** *P <* 0.0001 by unpaired t-test with Welch's correction, error bars = SEM.

To reliably model drug delivery in AD, we generated iBECs from previously published *APOE3* and *APOE4*-carrying human iPSCs (N = 3), including one isogenic pair in which both *APOE4* alleles had been converted to *APOE3* using CRISPR-Cas9 37-39. iBECs were generated using a previously published protocol 18 and differentiation confirmed by the presence of BBB markers occludin, claudin-5 and ZO-1 well as the formation of a characteristic cobblestone-like morphology in each line (Figure 1A and Figure S1). To ensure physiologically relevant barrier integrity, TEER was measured in Transwells containing a membrane with pores of 0.4 μm in diameter (Ø) - a Transwell format most commonly used in iBEC-based models 18, 40, 41. iBECs from all lines readily formed cobblestone-like monolayers on Ø 0.4 μm pore Transwells, however, *APOE4* iBECs demonstrated significantly reduced TEER (*P <* 0.0001) compared to *APOE3* iBECs (1985 ± 181 *vs*. 3056 ± 66 Ohm x cm^2^, respectively, mean ± SE; Figure 1B), with the TEER of both iBEC groups falling within the *in vivo* range of 1000 - 5900 Ohm x cm^2^ (reviewed in 42, 43). *APOE4* iBECs also exhibited a higher variation in TEER measurements than *APOE3* lines, which clustered more closely together (Figure 1B). To assess passive permeability of the formed barrier, leakage of fluorescently-conjugated 5 kDa dextran across the iBEC monolayer was measured, with significantly increased dextran permeability (*P =* 0.0022) observed in *APOE4* iBECs compared to *APOE3* iBECs, indicating reduced barrier integrity of *APOE4* iBECs (Figure 1B). To ensure differences in TEER and permeability were not caused by inefficient differentiation, we measured the relative gene expression of BBB markers occludin (*OCLN*), claudin-5 (*CLDN5*)*,* ZO-1 (*TJP1*)*,* VE-cadherin (*CDH5*) as well as the endothelial cell-specific SRY-box transcription factor 18 (*SOX18*) (Figure 1C). Compared to undifferentiated iPSCs, the expression of all studied junctional markers was significantly upregulated (*P* < 0.05) in *APOE3* and *APOE4* iBECs, with *SOX18* also being significantly increased (*P* = 0.0196) in *APOE3* iBECs compared to iPSC lines and showing a trend towards an increase in *APOE4* iBECs (Figure 1C). When the gene expression levels of the studied markers were compared between *APOE3* and *APOE4* iBECs, some significant differences were identified, with *CLDN5* (*P* = 0.021), *CDH5* (*P =* 0.003) and *SOX18* (*P =* 0.0495) expressed at a significantly higher level in *APOE4* iBECs compared to *APOE3* iBECs. This suggests a potential influence of the *APOE4/4* genotype on BEC phenotype (Figure 1D).

### RNA sequencing reveals lack of transcriptome changes in *APOE3* and* APOE4* iBECs following FUS^+MB^

Previous animal studies have identified changes in the microvascular transcriptome following FUS^+MB^ 44; however, molecular responses of the human BBB to FUS^+MB^ are largely unknown. Therefore, we performed bulk RNA-sequencing (RNA-seq) on FUS^+MB^-treated *APOE3* and *E4* iBECs. To achieve this, *APOE3* and *E4* iBECs were treated with FUS^+MB^ and collected at 1 h and 24 h post sonication, analogous to the previously reported timeline of BBB opening and spontaneous closing post FUS^+MB^ in *in vitro* models 18, 45. Corresponding untreated (UT) controls at the respective timepoints were included.

**Figure 2:**
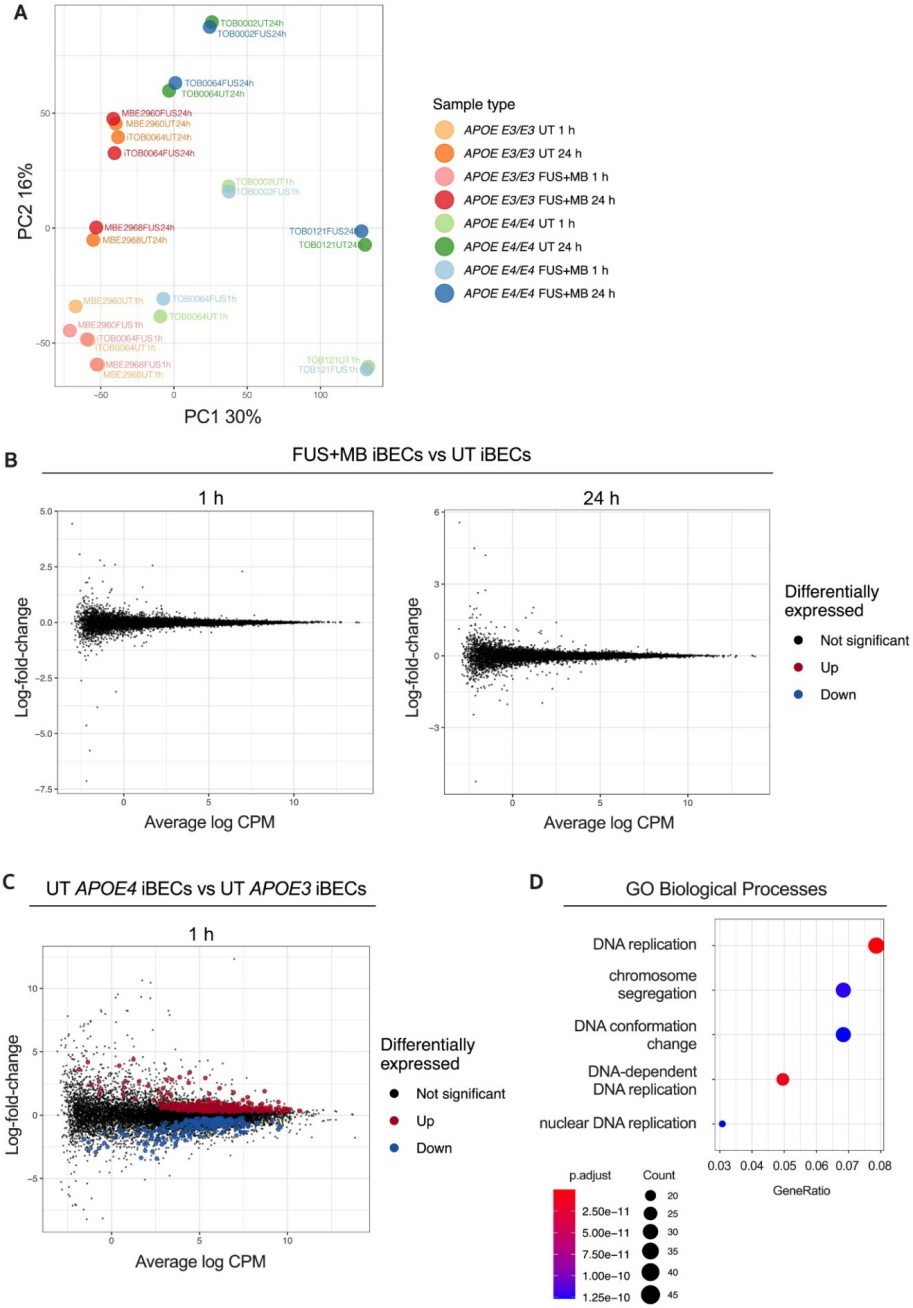
**Transcriptome analysis of the response to FUS^+MB^ in *APOE3* and *APOE4* iBECs**. (**A**) Principal component analysis (PCA) of gene expression profiles for all iBEC samples. The percentage of variance explained by principal component 1 (PC1) and principal component 2 (PC2) shown in axis labels. (**B**) Mean-difference (MD) plots showing log-fold-change vs average log expression values (log2 counts per million, CPM). Left panel: FUS^+MB^ treated *APOE3* and *APOE4* iBECs at 1 h vs UT *APOE3* and *APOE4* iBECs at 1 h. Right panel: FUS^+MB^ treated *APOE3* and *APOE4* iBECs at 24 h vs UT *APOE3* and *APOE4* iBECs at 24 h. (**C**) MD plot showing log-fold-change and average log expression values in UT *APOE4* iBECs at 1 h versus *APOE3* iBECs at 1 h. N = 3 biological replicates. (**D**) Dot plot of top 5 gene ontology (GO) terms from sub ontology Biological Process enriched from comparison of UT *APOE4* iBECs at 1h vs UT *APOE3* iBECs at 1 h. The dot size represents the number of genes associated with the GO term and the dot color represents the FDR value. The differentially expressed genes (i.e. FDR < 0.05) from this comparison were used as input to the analysis.

To gain insight into the relationships between the analyzed samples, we first performed Principal Component Analysis (PCA) on our RNA-seq dataset (Figure 2A). This revealed that the largest variation between the samples was associated with *APOE3* and *APOE4* genotype (principal component 1, PC1) and sample collection time (PC2). Surprisingly, UT and FUS^+MB^-treated samples largely clustered together in donor pairs, providing the first indication that FUS^+MB^ had little effect on the iBEC transcriptome. Indeed, differential expression analysis identified no differentially expressed genes (DEGs) between FUS^+MB^ and UT at either timepoint analyzed irrespective of the *APOE* genotype (Figure 2B), suggesting that the effects of FUS^+MB^ on gene expression in human iBECs are minimal. Gene expression also did not differ when the *APOE* genotype was incorporated into our analysis (Figure S2), indicating that the presence of *APOE E3* or *E4* allele did not have a profound effect on iBEC responses to FUS^+MB^. We then compared UT *APOE3* and UT *APOEE4* iBECs at 1 h which interestingly revealed 635 DEGs (FDR < 0.05), with 405 significantly up-regulated and 230 significantly down-regulated genes in *APOE4* iBECs compared to *APOE3* iBECs (Figure 2C, Table S1). Intriguingly, several genes identified as the most up- or down-regulated in *APOE4* iBECs, including *SHMT1* 46, *HMGB2* 47, *ALDH7A1* 48, *ABL2* 49, have previously been linked to cognitive dysfunction and AD, although their role at the BBB requires further investigation. Finally, to explore the biological function of identified DEGs between UT *APOE4* and *APOE3* iBECs at 1 h, we conducted Gene Ontology (GO) enrichment analysis for biological processes. The results revealed that the DEGs were significantly enriched in 302 GO terms (Table S2) with the top three enriched terms being “DNA replication”, “DNA-dependent DNA replication” and “chromosome segregation” (Figure 2D, Figure S3) suggesting alterations in DNA and cell division processes in *APOE4* iBECs.

### FUS^+MB^ increases the *in vitro* delivery of potentially therapeutic Alzheimer's antibodies Aducanumab and anti-Tau (RNF5)

**Figure 3:**
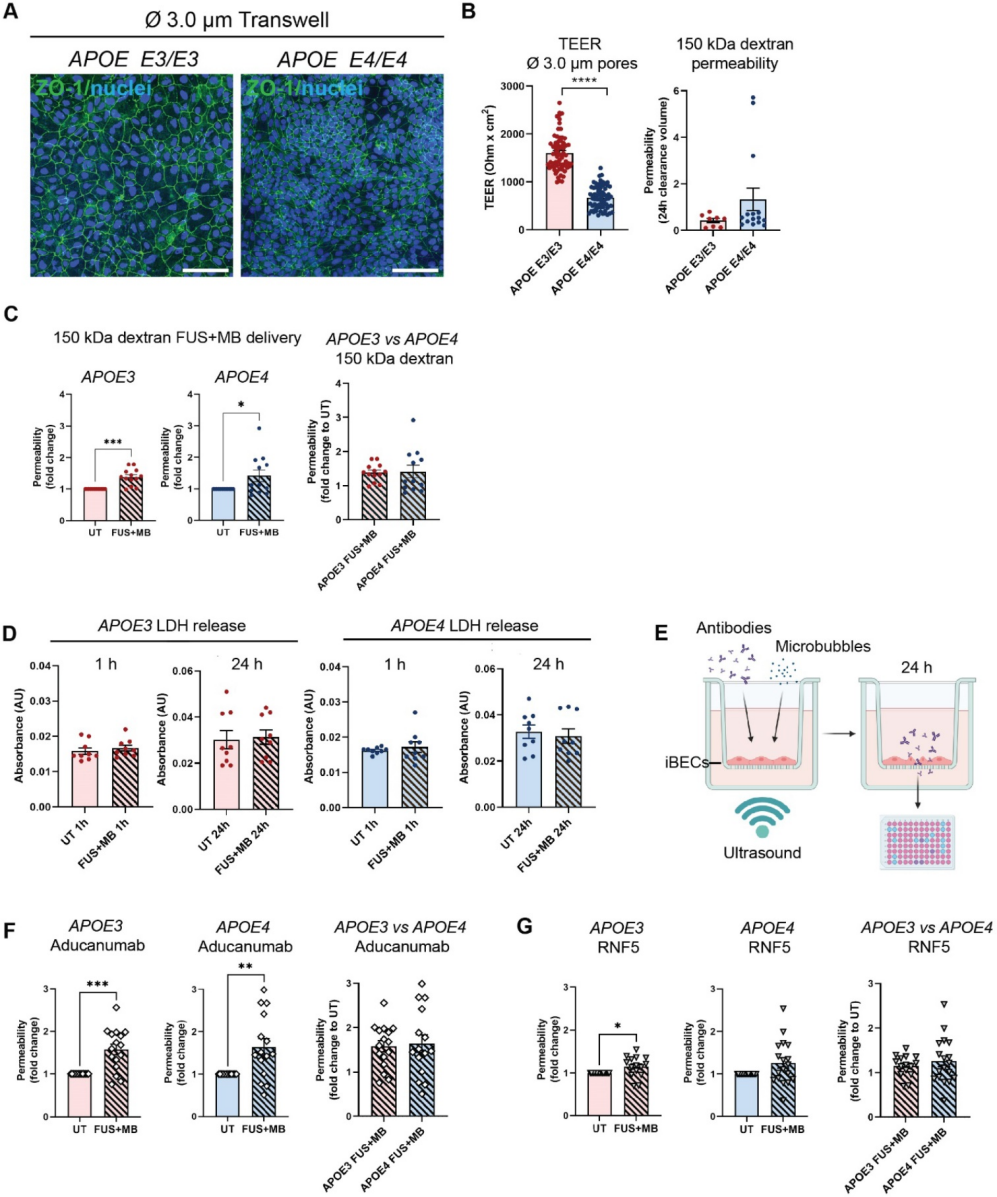
**Therapeutic antibody delivery in *APOE3* and *APOE4* iBEC monocultures following FUS^+MB^.** (**A**) Immunofluorescence images of iBECs cultured in Ø 3.0 µm Transwell stained with ZO-1 (green, Hoechst counterstain, scale bar = 100 µm). (**B**) Trans-endothelial electrical resistance (TEER, Ohm x cm^2^) and passive 150 kDa dextran permeability (24 h clearance volume) in *APOE3* and *APOE4* iBECs in 3.0 µm Transwells. (**C**) Delivery of 150 kDa dextran in *APOE3* and *APOE4* iBECs using focused ultrasound + microbubbles (FUS^+MB^). Permeability shown as fold change to untreated (UT) at 24 h. (**D**) Lactate dehydrogenase (LDH) release (shown as absorbance, AU) in UT and FUS^+MB^ treated *APOE3* and *APOE4* iBECs at 1 h and 24 h following treatment. (**E**) Schematic illustration of therapeutic antibody delivery using FUS^+MB^ in the Transwell system. Fluorescently-conjugated antibodies were added together with MBs to a Transwell containing iBECs and the insert was exposed to ultrasound. Flow-through of antibodies was measured 24 h following treatment using a fluorescent plate reader. Graphic created using Biorender.com. (**F**) Aducanumab-analogue delivery in UT and FUS^+MB^ treated *APOE3* and *APOE4* iBECs as well as comparison of Aducanumab delivery in FUS^+MB^ treated *APOE3* vs *APOE4* iBECs (permeability shown as relative values to UT at 24 h). (**G**) RNF5 delivery in UT and FUS^+MB^ treated *APOE3* and *APOE4* iBECs, as well as comparison of RNF5 delivery in FUS^+MB^ treated *APOE3* vs *APOE4* iBECs (permeability shown as relative values to UT at 24 h). N = 3 biological replicates and minimum n = 3 independent replicates per cell line. * *P <* 0.05, ** *P <* 0.01, *** *P <* 0.001, **** *P <* 0.0001 by unpaired t-test with Welch's correction or by one-sample t-test (for fold change values where UT = 1), error bars = SEM.

Although the classically used Ø 0.4 µm pore Transwells when coated with collagen IV and fibronectin (in the absence of iBECs) readily allow the passage of small molecule (5 kDa) dextran, our preliminary experiments revealed that this format of inserts drastically limits the permeability of the large molecule (150 kDa) dextran, which corresponds to therapeutic antibody size. To enable increased antibody delivery through the Transwell membrane itself, we established a new Transwell model whereby iBECs were cultured on wider, Ø 3.0 µm pore Transwell inserts with the use of these inserts alone (containing collagen IV and fibronectin coating but no iBECs) resulting in a 7.5 fold increase in 150 kDa dextran transfer through the membrane compared to Ø 0.4 μm pore inserts (Figure S4A). By further increasing the seeding cell number by 1.5 fold, compared to the cell number used for the Ø 0.4 μm pore Transwells, we were also able to generate classical 'cobblestone' forming iBEC monolayers in Ø 3.0 μm pore inserts assessed by ZO-1 localization to TJs (Figure 3A and Figure S4B). In addition, we were able to obtain TEER values for *APOE3* (1609 ± 46 Ohm x cm^2^, mean ± SE) and *APOE4* iBECs (672 ± 29 Ohm x cm^2^, mean ± SE), which, albeit lower than in Ø 0.4 μm pore Transwells, were still substantially higher than those reported for any primary or immortalized BEC-based Transwell model 42. Consistent with the findings in Ø 0.4 μm pore Transwells reported above, TEER values in Ø 3.0 μm pore Transwells were significantly lower (*P <* 0.0001) for *APOE4* iBECs compared to *APOE3* iBECs with *APOE4* iBECs showing a trend towards increased passive permeability to 150 kDa dextran (Figure 3B).

Using previously established FUS^+MB^ parameters 18, which are also clinically relevant (summarized in 50), we next assessed 150 kDa dextran permeability following exposure to FUS^+MB^. When FUS^+MB^ was applied to *APOE3* and *APOE4* iBEC monolayers cultured on Ø 3.0 μm pore Transwells, significantly increased delivery of 150 kDa dextran was observed compared to untreated (UT) cells in both cell groups (*APOE3 P =* 0.0002; *APOE4 P =* 0.0469) (Figure 3C). No differences were identified in FUS^+MB^-mediated dextran delivery efficiency between *APOE3* and *APOE4* cells (Figure 3C). To confirm that the effect of FUS^+MB^ on iBEC monolayer integrity was transient and not caused by FUS-alone without MBs (FUS^only^), we performed TEER and 5 kDa dextran permeability analysis on UT, FUS^only^ and FUS^+MB^ conditions in *APOE3* and *APOE4* cultures at 1 h and 24 h following treatment. The results demonstrated that TEER was significantly reduced (*P <* 0.0001) compared to UT 1 h post FUS^+MB^ (and not FUS^only^), with TEER fully recovered at 24 h, suggesting a transient effect of FUS^+MB^ on iBEC monolayer integrity, consistent with our previous reports 18 (Figure S4C). For 5 kDa dextran permeability analysis, we first analyzed dextran permeability 1 h following treatment, then removed the dextran and re-applied it at 24 h following treatment to examine barrier closure. Similarly to TEER, dextran permeability was significantly increased (*P <* 0.01) 1 h following FUS^+MB^ compared to UT in both *APOE3* and *APOE4* cells, while there was no effect with FUS^only^ (Figure S4D). Interestingly, at 24 h following treatment, there were no differences in dextran permeability between the treatment conditions in *APOE3* cells, indicating monolayer recovery (Figure S4D). However, *APOE4* cells continued to display increased dextran permeability when examined 24 h following FUS^+MB^ (Figure S4D). These results indicate potentially slower recovery of *APOE4* iBECs to FUS^+MB^, consistent with what we have previously reported for familial AD iBECs 18. Also, when FUS^+MB^-mediated reduction in TEER and increase in 5 kDa dextran permeability were compared between *APOE3* and *APOE4* iBECs at the 1 h timepoint, *APOE4* iBECs demonstrated stronger responses compared to *APOE3* cells, indicating potential disease risk-related responses to FUS^+MB^ (Figure S4E).To assess the effects of FUS^+MB^ on cell viability, we used a lactate dehydrogenase (LDH) cytotoxicity assay; however, we did not detect differences in LDH levels between samples collected from UT and FUS^+MB^ treated cells in *APOE3* or *APOE4* iBECs, suggesting that the effects of FUS^+MB^ on iBEC monolayer integrity were not caused by cell death (Figure 3D).

Following optimization of the 3.0 μm Transwell platform for FUS^+MB^-mediated drug delivery, we proceeded to measure the delivery of a fluorescently-conjugated Aducanumab-analogue (anti-amyloid-β) and RNF5 (anti-Tau) therapeutic antibodies in our model system. Briefly, 48 h following purification on 3.0 μm Transwells, iBECs were simultaneously treated with 1 μM (150 μg/ml) of the antibody of interest along with MBs and exposed to FUS at 0.3 MPa (286 kHz center frequency) for 120 s (Figure 3E). The utilized antibody concentration correlated with previous *in vitro* studies using therapeutic antibodies 51-55. Fluorescence of the flow-through, indicating antibody delivery, was measured 24 h following treatment (Figure 3E). Interestingly, Aducanumab delivery was significantly increased in both *APOE3* (1.58 ± 0.12 fold, mean ± SE, *P =* 0.0003) and *APOE4* (1.64 ± 0.2 fold, mean ± SE, *P =* 0.0059) iBEC cultures following FUS^+MB^ treatment compared to UT cells (Figure 3F). The delivery efficiency of Aducanumab following FUS^+MB^ was not significantly different between* APOE3* and *APOE4* iBECs. Notably, the delivery of RNF5 was significantly increased in *APOE3* (1.15 ± 0.06 fold, mean ± SE, *P =* 0.0295) with *APOE4* iBECs demonstrating a strong trend toward an increase in RNF5 delivery (1.26 ± 0.12 fold, mean ± SE, *P =* 0.0501) when treated with FUS^+MB^ as compared to UT (Figure 3G). Similar to Aducanumab, RNF5 delivery in FUS^+MB^ treated *APOE3* and *APOE4* iBECs did not significantly differ (Figure 3G). Interestingly, when the delivery efficiency of the two antibodies was compared in each cell group, Aducanumab delivery following FUS^+MB^ treatment was significantly higher (*P =* 0.0052) than RNF5 in *APOE3* iBECs, while in *APOE4* iBECs there was no significant difference in the delivery efficiency of the two antibodies (Figure S4F).

### FUS^+MB^ treatment has minimal effects on *APOE3* and *APOE4* iAstrocyte phenotype

Astrocytes are a critical component of the BBB, but little is known about the effects of FUS^+MB^ on human astrocytes. As astrocyte end-feet are in direct contact with BECs *in vivo*, it is likely that FUS^+MB^ treatment also impacts astrocytes, however, this has not been extensively investigated. We therefore examined astrocyte responses to FUS^+MB^ within the first 24 h following treatment. We generated induced astrocytes (iAstrocytes) from the same *APOE3* and *APOE4*-carrying human iPSCs that were used for iBEC differentiation (n=2 biological replicates including 1 isogenic pair). Neural progenitor cells (NPCs) were generated and characterized for nestin and SOX2 expression (Figure S5A) and then differentiated into iAstrocytes for a minimum of 60 days. iAstrocytes were further matured 7 days before experiments with 10 ng/mL bone morphogenetic protein 4 (BMP-4) and ciliary neurotrophic factor (CNTF) as previously described 56. Matured iAstrocytes were exposed to FUS^+MB^ using the same parameters as for therapeutic antibody delivery in iBEC monocultures, and cell morphology, viability, marker expression and inflammatory responses were examined 1 h and 24 h following treatment.

**Figure 4:**
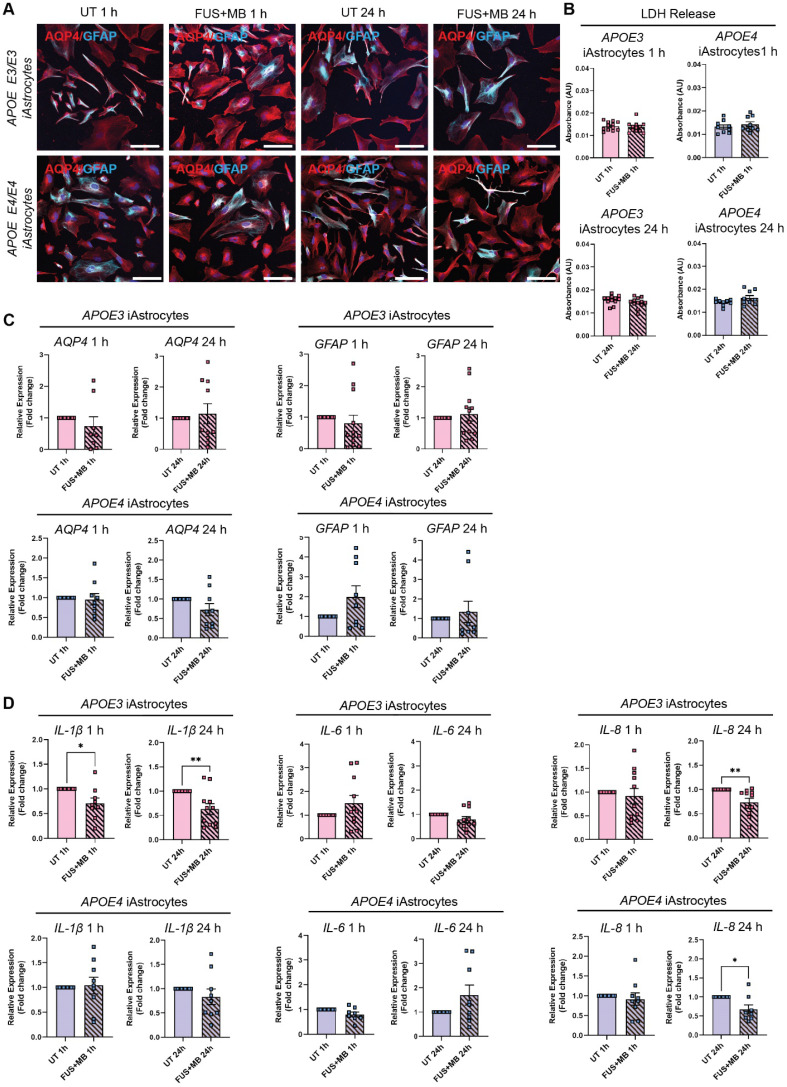
**Effect of FUS^+MB^ on *APOE3* and *APOE4* iAstrocytes** (**A**) Representative immunofluorescence images of *APOE3* and *APOE4* iAstrocytes in untreated (UT) and focused ultrasound + microbubble (FUS^+MB^) conditions 1 h and 24 h following treatment stained with AQP4 (red) and GFAP (cyan; Hoechst counterstain, scale bar = 100 µm). (**B**) Lactate dehydrogenase (LDH) release (shown as absorbance, AU) in UT and FUS^+MB^ treated *APOE3* and *APOE4* iAstrocytes 1 h and 24 h following treatment. (**C**) Relative gene expression of astrocyte markers *AQP4* and *GFAP* in UT and FUS^+MB^ treated *APOE3* and *APOE4* iAstrocytes 1 h and 24 h after treatment. (**D**) Relative gene expression of inflammatory markers *IL-1β*, *IL-8* and *IL-6* in UT and FUS^+MB^ treated *APOE3* and *APOE4* iAstrocytes 1 h and 24 h after treatment. N = 2 biological replicates and minimum n = 3 independent replicates per line. * *P <* 0.05, ** *P <* 0.01 by unpaired t-test with Welch's correction or by one-sample t-test (fold change values), error bars = SEM.

Following FUS^+MB^ treatment, *APOE3* and *APOE4* iAstrocytes did not display any adverse morphological changes compared to UT cells when stained with astrocyte markers aquaporin-4 (AQP4) and glial fibrillary acidic protein (GFAP) 1 h and 24 h following treatment (Figure 4A and Figure S5B). In addition, the LDH assay did not reveal any significant changes in cell viability between UT and FUS^+MB^ conditions at 1 h and 24 h timepoints in *APOE3* or *APOE4* iAstrocytes (Figure 4B). Next, we analyzed the relative gene expression of astrocyte markers following FUS^+MB^ treatment. No significant changes in expression of astrocyte markers *AQP4*, *GFAP* (Figure 4C) and *S100B* (Figure S6A) were identified in FUS^+MB^ treated *APOE3* or *APOE4* iAstrocytes compared to UT iAstrocytes. Following this, to identify whether FUS^+MB^ elicited any inflammatory responses in iAstrocytes, we examined the gene expression of inflammatory cytokines known to be secreted by iPSC-derived astrocytes in basal culture conditions and following inflammatory stimulation 57-59. Interestingly, interleukin-1β (*IL-1β*) expression was significantly reduced in *APOE3* iAstrocytes 1 h (*P =* 0.028) and 24 h (*P =* 0.009) following FUS^+MB^ treatment, whereas in *APOE4* iAstrocytes its expression was not altered (Figure 4D). Similarly, *IL-6* and *IL-8* expression levels were not altered in *APOE3* or *APOE4* iAstrocytes 1 h following FUS^+MB^, however, interestingly the expression levels of *IL-8* were significantly downregulated in *APOE3* (*P =* 0.008) and *APOE4* (*P* = 0.024) iAstrocytes 24 h following FUS^+MB^ treatment (Figure 4D). No changes were identified in the expression of C-C motif chemokine ligand 2 (*CCL2*), an astrocytic chemokine acting as an important mediator of damage-associated neuroinflammation 60, 61, in *APOE3* or *APOE4* following FUS^+MB^ treatment for either timepoint (Figure S6B). Finally, we compared cytokine expression in a FUS^+MB^-treated isogenic *APOE3*/*APOE4* iAstrocyte pair (parent *APOE4* iPSC line converted to *APOE3* (*iAPOE3*) 37). Interestingly, the results demonstrated a decreased inflammatory response in *iAPOE3* iAstrocytes compared to *APOE4* cells 1 h after treatment, with significantly decreased *IL-1β* (*P =* 0.025) expression and *CCL2* demonstrating a trend in decreased expression (*P =* 0.0501) in i*APOE3* iAstrocytes (Figure S6C). At 24 h, these differences were no longer evident, though *IL-8* expression was significantly decreased (*P* = 0.047) in *APOE4* vs *iAPOE3* iAstrocytes.

### iBECs co-cultured with iAstrocytes demonstrate increased barrier integrity and allow therapeutic antibody delivery following FUS^+MB^

**Figure 5:**
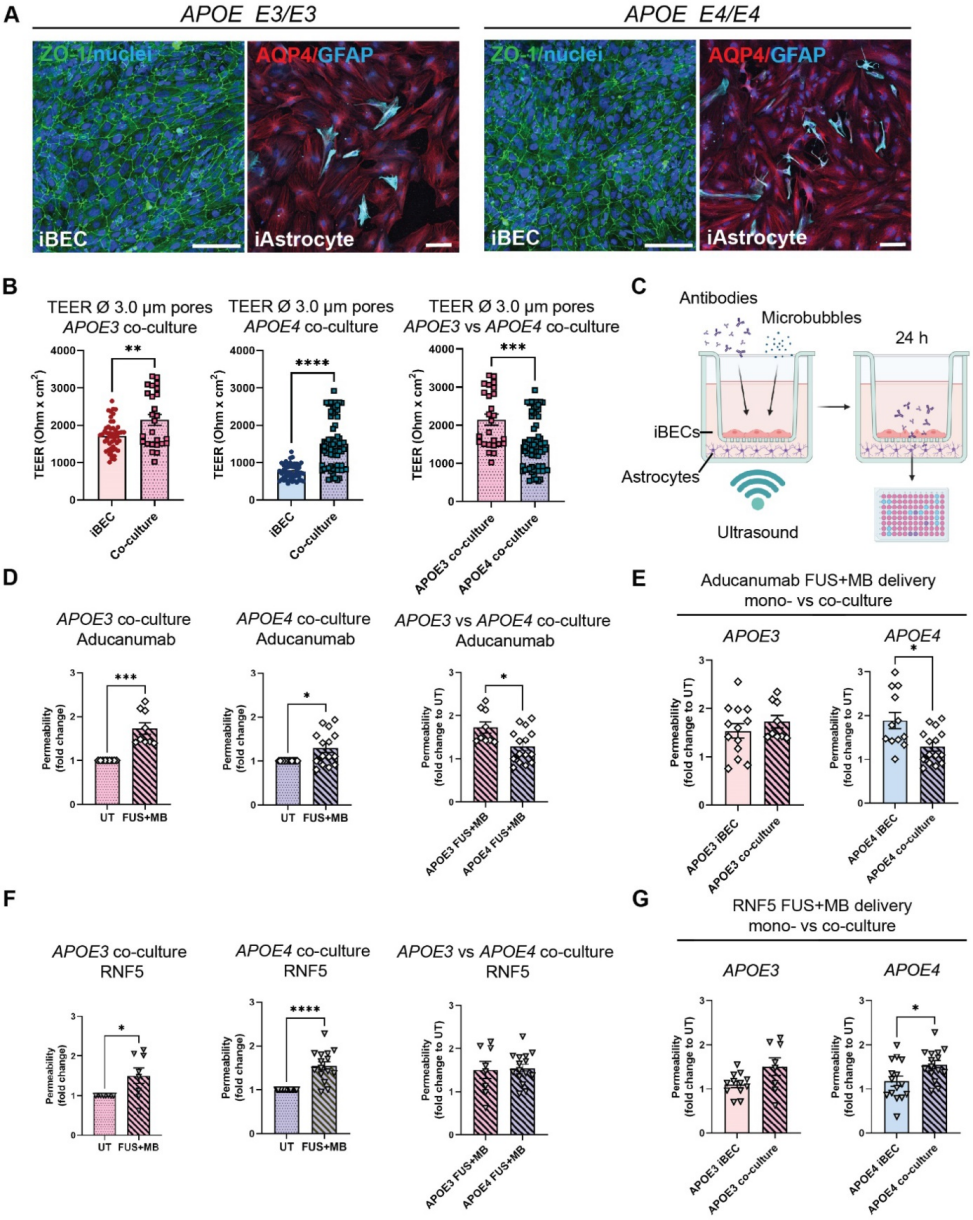
**Therapeutic AD antibody delivery in *APOE3* and *APOE4* iBEC+iAstrocyte co-cultures following FUS^+MB^.** (**A**) Representative immunofluorescence images of *APOE3* and *APOE4* iBECs (ZO-1/green) and iAstrocytes (AQP4/red, GFAP/cyan) in co-culture, maintained in endothelial serum free medium + B-27 (Hoechst counterstain, scale bar = 100 µm). (**B**) Comparison of trans-endothelial electrical resistance (TEER, Ohm x cm^2^) in *APOE3* and *APOE4* iBECs and iBEC+astrocyte co-cultures as well as *APOE3* vs *APOE4* co-cultures in Ø 3.0 μm Transwells. (**C**) Schematic illustration of therapeutic antibody delivery using FUS^+MB^ in iBEC+iAstrocyte co-cultures. iBECs+iAstrocytes were co-cultured in the Transwell system for 24 h after which antibodies were added together with MBs and the insert exposed to ultrasound. Flow-through of antibodies was measured 24 h following treatment using a fluorescence plate reader. Graphic created using Biorender.com. (**D**) Aducanumab-analogue delivery in UT and FUS^+MB^ treated *APOE3* and *APOE4* iBEC+iAstrocyte co-cultures, as well as comparison of Aducanumab delivery in FUS^+MB^ treated *APOE3* vs *APOE4* iBEC+iAstrocyte co-cultures. Permeability shown as relative values to UT at 24 h. (**E**) Comparison of Aducanumab delivery following FUS^+MB^ in *APOE3* and *APOE4* iBEC mono- and iBEC+iAstrocyte co-cultures (permeability shown as relative values to UT at 24 h). (**F**) RNF5 delivery in UT and FUS^+MB^ treated *APOE3* and *APOE4* iBEC+iAstrocyte co-cultures, as well as comparison of RNF5 delivery in FUS^+MB^ treated *APOE3* vs *APOE4* iBEC+iAstrocyte co-cultures. Permeability shown as relative values to UT at 24 h. (**G**) Comparison of RNF5 delivery following FUS^+MB^ in *APOE3* and *APOE4* iBEC mono- and iBEC+iAstrocyte co-cultures (permeability shown as relative values to UT at 24 h). N = 2 biological replicates and minimum n = 3 independent replicates per cell line. * *P <* 0.05, *** *P <* 0.001, **** *P <* 0.0001 by unpaired t-test with Welch's correction or by one-sample t-test (fold change values), error bars = SEM.

As a step toward developing a multicellular AD BBB model for FUS^+MB^ mediated therapeutic antibody delivery, we next established iBEC+iAstrocyte co-cultures in Ø 3.0 μm pore Transwells as astrocytes are known to be key players in enhancing BBB integrity 62. Cells were co-cultured for 24 h prior to performing antibody delivery experiments. With sample collection performed 24 h following treatment, the total time in co-culture was 48 h. Both cell types were maintained in endothelial serum-free medium (ESFM) + B+27 supplement, with the co-culture conditions supporting normal marker expression of both cell types (Figure 5A). Twenty-four hours following co-culture, TEER was significantly increased in both *APOE3* (2147 ± 145 Ohm x cm^2^, mean ± SE,* P =* 0.0082) and *APOE4* (1504 ± 80 Ohm x cm^2^, mean ± SE, *P <* 0.0001) co-cultures when compared to the respective iBEC monocultures (*APOE3* iBECs: 1609 ± 46 Ohm x cm^2^, mean ± SE; *APOE4* iBECs: 672 ± 29 Ohm x cm^2^, mean ± SE; Figure 3B) in Ø 3.0 μm pore Transwells (Figure 5B). This supports barrier enhancing effects of iAstrocytes. Interestingly, when we compared TEER values between *APOE3* and *APOE4* co-cultures, TEER was significantly reduced (*P =* 0.0004) in *APOE4* co-cultures compared to *APOE3* co-cultures, in line with our findings from iBEC monoculture experiments. Next, we performed therapeutic AD antibody delivery in iBEC+iAstrocyte co-cultures with FUS^+MB^ and measured delivery efficiency 24 h following treatment (Figure 5C). Similar to iBEC monocultures, Aducanumab delivery was significantly increased in both *APOE3* (1.73 ± 0.39 fold, mean ± SE, *P =* 0.0005) and *APOE4* (1.29 ± 0.1, mean ± SE, *P =* 0.012) co-cultures (Figure 5D). Interestingly, the delivery of Aducanumab following FUS^+MB^ was significantly lower (*P* = 0.017) in* APOE4* co-cultures compared to *APOE3* co-cultures (Figure 5D). When compared to iBEC monocultures, there was no difference in Aducanumab delivery efficiency in *APOE3* co-cultures, while in contrast, *APOE4* co-cultures demonstrated significantly lower (*P =* 0.012) Aducanumab delivery when compared to *APOE4* monocultures (Figure 5E). RNF5 delivery following FUS^+MB^ was next investigated in *APOE3* and *APOE4* co-cultures, and delivery was significantly increased in both culture types compared to UT (*APOE3*: 1.50 ± 0.2 fold, *P =* 0.038; *APOE4* 1.55 ± 0.09 fold, *P <* 0.0001, mean ± SE) with no significant difference in RNF5 delivery between *APOE3* and *APOE4* co-cultures observed (Figure 5F). As for Aducanumab, the delivery efficiency of RNF5 was similar between *APOE3* co- and iBEC monocultures following FUS^+MB^ (Figure 5G). In contrast, compared to iBEC monocultures, RNF5 delivery efficiency following FUS^+MB^ was significantly increased (*P =* 0.018) in *APOE4* co-cultures, suggesting possible modifying effects of iAstrocytes (Figure 5G). There was no difference in the delivery efficiency of Aducanumab and RNF5 following FUS^+MB^ within *APOE3* or *APOE4* co-culture groups (Figure S7).

### A novel hydrogel-based 2.5D BBB model provides a physiologically relevant alternative to the Transwell system for high-throughput drug delivery studies

**Figure 6:**
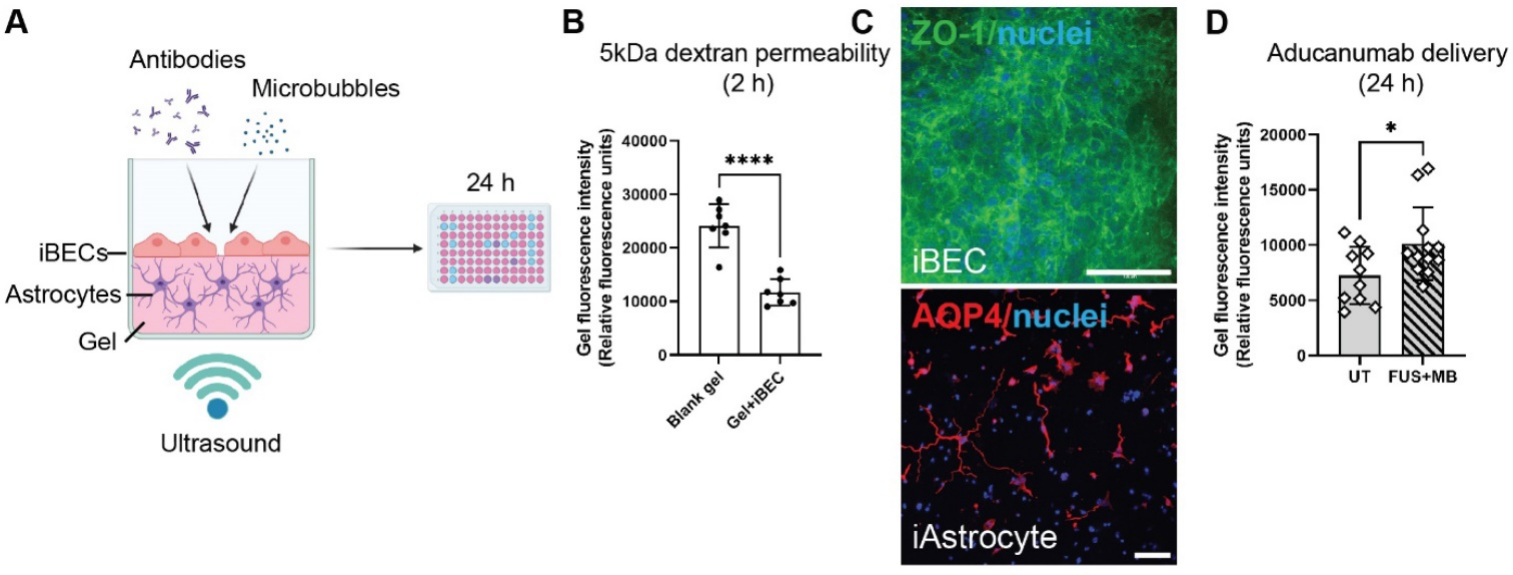
**A 2.5D gel-based BBB model for screening of FUS^+MB^-mediated antibody delivery.** (**A**) A schematic illustration of therapeutic antibody delivery using FUS^+MB^ in LunaGel^TM^-based 2.5D model. iAstrocytes were embedded in the gel and allowed to mature for 7 days. Following purification yielding a highly homogeneous population of iBECs, the cells were seeded on top of the iAstrocyte layer and exposed to therapeutic antibodies, MB and ultrasound. Fluorescence intensity within the gel, indicating antibody delivery, was measured 24 h following treatment using a fluorescence plate reader. Graphic created using Biorender.com. (**B**) Passive dextran permeability shown as relative fluorescence units in a blank (no cell containing) LunaGel^TM^ and an iBEC containing LunaGel^TM^ measured at 2 h after exposure. (**C**) Representative immunofluorescence images of iBECs (ZO1/green, 40x magnification) and iAstrocytes (10x magnification) in the LunaGel^TM^ platform. Hoechst counterstain, scale bar = 100 μm. (**D**) Aducanumab-analogue delivery in UT and FUS^+MB^ treated iBEC+iAstrocyte 2.5D model indicated as relative fluorescence units. N = 3 iPSC lines. * *P <* 0.05, by unpaired t-test with Welch's correction, error bars = SEM.

While the Transwell system for drug permeability screening is easy to establish and widely used, it requires large volumes of reagents (such as culture media and antibodies). The biomimicry of the system is also inhibited by the use of an artificial membrane to co-culture cells. Additionally, the inherent 6- to 24-well format of the Transwell system limits its use for high-throughput drug screening. To overcome these issues, we developed a 2.5D gel-based BBB-like model in a 96-well plate format that could offer a more physiologically relevant alternative. Here, astrocytes are grown in 3D and iBECs in 2D, and are in contact with each other without a separating membrane (Figure 6A). In addition, the reagent requirements for high-throughput screening of antibodies or alternative drug delivery methods are downscaled in this model. Matrigel and collagen I are commonly used for 3D assays, however, they often display batch-to-batch variability and are too soft for establishing a BBB model. Thus, we trialed LunaGel^TM^, a gelatin-based photocrosslinkable gel, which can be adjusted to a desired stiffness using varying polymerization times under blue light (Gelomics). Briefly, to establish a 3D culture we first embedded iAstrocytes in LunaGel^TM^ and matured iAstrocytes for 7 days. Next, iBECs were purified on collagen IV and fibronectin yielding a highly homogeneous BEC-like population as shown by us and others 18, 40. The iBECs were subsequently seeded in 2D on top of the iAstrocyte layer, on a new layer of high-stiffness LunaGel^TM^ and allowed to attach for 24 h. The 2.5D co-culture model was then exposed to antibodies and FUS^+MB^ as in the Transwell system and fluorescence intensity, indicative of drug delivery, was measured in the gel 24 h following treatment using a plate reader (Figure 6A). Prior to establishing co-cultures, iBEC barrier formation on the layered LunaGel^TM^ was confirmed by performing passive 5 kDa FITC-conjugated dextran permeability analysis. Our results demonstrated that the presence of iBECs significantly (*P <* 0.0001) hindered dextran delivery into the gel by over 2-fold compared to the blank gel (without iBECs), indicating barrier formation (Figure 6B). Barrier formation of iBECs on the LunaGel^TM^ was further confirmed by ZO-1 staining, which showed localization to cell-cell junctions (Figure 6C, enlarged grayscale images shown in Figure S8). Interestingly, our results also demonstrated that iAstrocytes readily proliferated and extended their processes within the low stiffness LunaGel^TM^ gel (Figure 6C). Intriguingly, when exposing the 2.5D BBB model to FUS^+MB^, our initial observations have demonstrated significantly increased (*P =* 0.033) Aducanumab delivery (fold change 1.4 ± 0.12, mean ± SE), when compared to UT (Figure 6C). Although further optimization of this model is ongoing, our results highlight the potential of using a gel-based 2.5D model system for FUS^+MB^-mediated antibody delivery as a more physiologically relevant high-throughput platform with potentially higher translatability to patients than with the traditional Transwell system.

## Discussion

A major hindrance in treating neurodegenerative diseases such as AD is the low bioavailability of therapeutics in the brain due to the BBB, which inhibits the entry of most large molecule drugs, including antibodies. The FUS^+MB^ technique is a relatively novel technology that allows the transient opening of the BBB to achieve efficient drug delivery into the CNS 63. A small number of clinical studies using FUS^+MB^ in AD patients have demonstrated the safety of this technique 21, 23-25, making it a promising tool for increased drug delivery. However, our understanding of the molecular effects of FUS^+MB^ on human BBB cells is limited, and there is a lack of patient cell-based screening platforms to identify FUS^+MB^-deliverable drugs. Thus, patient cell models that recapitulate disease phenotype and allow for the testing of FUS^+MB^ effects are critical in accelerating the translation of FUS^+MB^ to the clinic for the treatment of a range of neurodegenerative diseases.

We have previously demonstrated the ability to increase BBB permeability and Aβ clearance using FUS^+MB^ in a patient cell Transwell model that incorporated iBECs carrying the familial AD *PSEN1* mutation 18. However, with familial AD accounting for less than 5% of all AD cases, this model is not highly representative of the majority of AD cases which are of sporadic origin. With the known association of the sporadic AD risk gene polymorphism *APOE4* on BBB dysfunction, we utilized *APOE4*-carrying patient-derived iPSCs to generate a BBB-like model to test the effects of FUS^+MB^ as well as the delivery of two therapeutic AD antibodies and compared the results to *APOE3* (normal AD risk control) cells. Our hypothesis was that a patient-specific sporadic AD cell model of the BBB will increase our understanding of FUS^+MB^ effects that are applicable to the majority of AD cases, thereby accelerating the translation of FUS^+MB^ into a clinical drug delivery method for the treatment of AD. Although questions have been raised about the accuracy of iPSC-derived iBECs in resembling *in vivo* BECs 64, these cells exhibit the highest TEER compared to any other BEC model making them ideal for mechanistic BBB opening studies such as FUS^+MB^ 16, 42. In addition, iBECs exhibit many properties of endogenous BECs and most closely resemble drug permeability characteristics of the human BBB when compared to other cell models 16, 41, further supporting their use. As such, we hypothesized that iBECs are the most appropriate cell model for FUS^+MB^-mediated drug delivery modelling *in vitro* in an AD context and opted for their use in this study.

Our characterization of *APOE3* and *APOE4* iBECs revealed several phenotypical differences that indicate the contribution of *APOE4* to BBB breakdown. Although both *APOE3* and *APOE4* iBECs generated a cobblestone-like morphology and expressed key BBB markers, the TEER of *APOE4* iBECs was significantly lower compared to *APOE3* iBECs in both mono- and iAstrocyte co-culture systems, supporting the association of *APOE4* with reduced BBB integrity. Reduced TEER observed in *APOE4* iBECs also correlated with increased permeability to a small molecule fluorescent tracer, suggesting the ability to model previously reported *APOE4*-induced BBB breakdown 11. We also identified some gene expression differences via RT-qPCR in BBB-associated TJs and AJs between *APOE3* and *APOE4* iBECs, suggesting potential modulatory effects of the *APOE4/4* genotype on BEC marker expression, correlating to previous reports in *APOE4* iPSC-derived endothelial cells 65 and our previous study using familial AD *PSEN1* mutation harboring iBECs 18. What the functional consequences of these expression differences are, remains to be elucidated.

As the molecular effects of FUS^+MB^ on human BBB cells are unknown, we investigated the iBEC transcriptome following FUS^+MB^ exposure. Intriguingly, we did not identify any genes that were significantly altered by FUS^+MB^ irrespective of disease phenotype. In line with our study, using single-cell RNA sequencing, Gorick and colleagues also detected only minimal changes (8 DEGs overall) in the transcriptome of the murine cerebrovascular endothelium when FUS^+MB^ was applied at low pressure (0.1, 0.2 and 0.4 MPa) 66. This suggests that FUS^+MB^ achieves the BBB opening by affecting junctional protein ultrastructure in BECs as previously suggested 67, 68, rather than by acting at the TJ or AJ gene expression level. However, further studies are required to confirm the mechanism behind FUS^+MB^-mediated barrier opening in the iBEC model. Interestingly, a previous study conducted in a murine model identified changes in the microvessel transcriptome following exposure to FUS^+MB^, pointing to a transient inflammatory response induced by sonication 44. Importantly, in contrast to our study, McMahon *et al*. 44 performed their analysis on dissected brain microvessels suggesting that the observed effects can be driven by other cells associated with brain microvasculature rather than just BECs. In support of this, previous studies have found effects of FUS^+MB^ on astrocyte and microglia activation 27, 69. The lack of changes in the iBEC transcriptome induced by FUS^+MB^ could also be explained by differences in FUS parameters and MB type/concentration applied compared to McMahon *et al*. and Gorick *et al*., illustrating the importance of better understanding the correlation between FUS and MB physical characteristics and cellular responses. Finally, interspecies differences reported in the transcriptome of murine and human microvessels 32, as well as high variability between patient-derived iPSC lines 37, could underlie the lack of finding changes in the transcriptome following FUS^+MB^. Overall, as we did not identify any adverse effects of FUS^+MB^ on iBEC viability nor effects on the iBEC transcriptome, we conclude that our study further supports the safety of FUS^+MB^ treatment.

Interestingly, our analysis identified over 600 DEGs in *APOE3* vs *APOE4* iBECs, and further analysis of these genes may potentially identify novel disease biomarkers. Correspondingly, our GO enrichment analysis suggests DNA replication and cell division changes in the *APOE4* iBECs. Intriguingly, defects in mitosis and chromosome segregation have been previously linked to AD pathogenesis, with *APOE4* being one of the suggested genetic drivers of this effect 70-74. Although alterations in BEC division have not yet been investigated in the context of BBB breakdown in AD, and only low levels of cell turnover have been previously reported in iBECs *in vitro* and BECs *in vivo* under normal conditions 75, 76, our observation might point to a novel pathomechanism driving BBB dysfunction in *APOE4* carriers.

Large molecule therapeutic antibodies are a promising treatment for various neurodegenerative diseases, but their size limits their effective delivery across the BBB into the brain. It is estimated that only 0.2% of the intravenously administered antibody concentration reaches the brain, severely limiting their therapeutic efficacy 77. As such, due to the BBB opening effects of FUS^+MB^ 23, 78, this technique provides a highly promising approach for large molecule therapeutic antibody delivery into the brain 26-28. With there being a lack of *in vitro* cell models for FUS^+MB^-mediated drug permeability screening, we tested our established *APOE3* and *APOE4* iBEC platform for FUS^+MB^-mediated AD antibody delivery using a similar approach to our previously published study 18. Our results confirmed the transient effects of FUS^+MB^ on iBEC monolayer integrity as shown by TEER, which was significantly reduced 1 h following treatment and fully recovered when assessed at the 24 h timepoint, correlating with our previous findings which also demonstrated recovery of TEER by 24 h following FUS^+MB^ 18. Interestingly, our results also suggest *APOE4* iBECs might be more susceptible to FUS^+MB^-mediated membrane disruption as these cells displayed higher reduction in TEER and slower recovery in barrier permeability, being in-line with our findings with familial AD iBECs in our previous study 18. Following the characterization of the effects of FUS^+MB^ on barrier integrity, our results demonstrated a significant increase in Aducanumab and RNF5 antibody delivery following FUS^+MB^ in both *APOE3* and *APOE4* iBEC mono- and co-culture models. This first-ever patient-derived cell-based model for FUS^+MB^-mediated therapeutic AD antibody delivery provides evidence of the ability to increase therapeutic antibody delivery using FUS^+MB^. Interestingly, no differences in antibody delivery efficiency were identified between *APOE3* and *APOE4* iBEC monocultures, suggesting that reduced BBB integrity associated with *APOE4* iBECs did not affect FUS^+MB^-mediated antibody delivery. However, in *APOE3* iBEC cultures FUS^+MB^-mediated delivery efficiency of Aducanumab was higher than RNF5, suggesting some antibodies might be more readily delivered using FUS^+MB^ than others, with patient-cell models potentially important in screening the FUS^+MB^-mediated delivery efficiency of drugs. In clinical trials, Aducanumab has been shown to significantly delay cognitive decline when administered at the highest possible dose 79. Thus, increasing the intracerebral concentration of Aducanumab with methods such as FUS^+MB^ is anticipated to improve clinical outcomes. In addition, since this study did not investigate whether FUS^+MB^-mediated antibody delivery occurs via the trans or paracellular routes, addressing the route of uptake in future studies will be an important step to better understand the modes of action of FUS^+MB^.

Astrocytes are central components of the BBB, enhancing barrier integrity and providing communication between the BBB and CNS 62. In addition, astrocytes are key mediators of inflammation as they secrete cytokines that in turn modulate other brain cells, such as microglia 80. Because the effects of FUS^+MB^ on human astrocytes have been sparsely investigated, we aimed to examine the potential modulatory effects of FUS^+MB^ on iAstrocytes and whether* APOE3* and *APOE4* iAstrocytes respond differently. Intriguingly, when iAstrocyte morphology, viability and marker expression was examined, we did not identify any modulatory effects of FUS^+MB^ within the 24 h timepoint examined. We also examined the gene expression of several cytokines known to be secreted by astrocytes under basal conditions and following inflammatory stimulation 57-59. Interestingly, our observations suggest that within the first 24 h following treatment, FUS^+MB^ does not induce an inflammatory response in iAstrocytes. In contrast, our results indicate that FUS^+MB^ may decrease the production of some inflammatory cytokines, with *IL-1β* and *IL-8* expression reduced at 1 h or 24 h following FUS^+MB^ treatment. Our results also suggest that *APOE4* isoform carrying iAstrocytes might be less susceptible to potential immunomodulatory effects of FUS^+MB^ with *IL-1β* expression not altered in these cells following treatment. Further experiments, such as the assessment of cytokine secretion following FUS^+MB^ will help to better elucidate any immunomodulatory effects of FUS^+MB^ on human astrocytes. In addition, since previous studies in mouse models have suggested that astrocyte activation following FUS^+MB^ might take several days 69, longer studies past the 24 h timepoint are needed in the human iPSC-derived BBB model to fully understand the effects of ultrasound on glial cells. Also, since blood-borne components are likely to enter the brain following FUS^+MB^ *in vivo*, the lack of these components in our model means that the full extent of astrocyte activation might not be evident *in vitro*. Thus, future studies should aim to incorporate blood-borne factors in the culture medium. Furthermore, other BBB cells, such as pericytes, also play a key inflammatory role in the BBB 81. As such, establishing a triple-culture model of BECs, astrocytes and pericytes is important for a complete BBB *in vitro* model to identify the full effects of FUS^+MB^.

Following characterization of FUS^+MB^ effects on iAstrocytes, we proceeded to establish iBEC+iAstrocyte co-cultures in an attempt to generate a more physiologically relevant BBB model for FUS^+MB^-mediated drug delivery screening. Consistent with previous studies 82, 83, co-culture of iBECs+iAstrocytes significantly increased barrier integrity in both *APOE3* and *APOE4* models. We then used the same parameters as for iBEC monocultures to deliver therapeutic AD antibodies in the co-culture systems. Supporting the reproducibility of our delivery system, we were able to significantly increase the delivery of both Aducanumab and RNF5 using FUS^+MB^ in the *APOE3* and *APOE4* co-culture systems. Interestingly, however, we observed some differences between *APOE3* and *APOE4* models in the co-culture systems, not identified in iBEC monocultures. One was that Aducanumab delivery efficiency in the *APOE4* co-culture system was lower compared to *APOE3* co-cultures and *APOE4* iBEC monocultures. In contrast, RNF5 delivery efficiency was significantly higher in *APOE4* co-cultures compared to *APOE4* monocultures, although TEER values in *APOE4* co-cultures were significantly higher. Without further investigation, it is difficult to hypothesize the physiological relevance of these observations in the human brain. One explanation could be that there are patient-specific differences in astrocyte function that modulate iBEC responses differently. Overall, the presence of astrocytes with iBECs is likely required in order to obtain a model that is more closely representative of the brain's BBB. Our established 2.5D BBB model further demonstrates the ability to culture iBECs and iAstrocytes in close contact within a supporting matrix and being able to use this model for FUS^+MB^-mediated drug delivery screening. Such a model, when fully optimized, will likely have higher translatability as a screening platform than traditional 2D or Transwell models.

Overall, our study presents a robust and reproducible BBB *in vitro* model to explore FUS^+MB^-mediated therapeutic antibody delivery with the ability to identify potential disease- or patient-specific differences in treatment response. Whilst the iPSC-derived cells used in this model provide advantages in terms of modelling human disease phenotypes, one limitation is that they are prone to high levels of inter-cell line variability. Therefore, it might be difficult to accurately identify disease-specific differences, unless the number of biological replicates is increased, particularly in sporadic AD. In addition, based on the *in vitro* results, it is difficult to predict how the increased antibody delivery following FUS^+MB^ would translate in clinic and whether the observed increases in antibody fold change would be large enough to substantially increase therapeutic efficacy. However, *in vivo* work in an AD mouse model has demonstrated a 5-fold increase in Aducanumab levels along with improvements in spatial memory following scanning ultrasound and MB treatment compared to passive antibody delivery, suggesting similar outcomes could be achieved in a human study 27. In addition, the physical dynamics of the free-floating MBs in our system may differ from those observed within the constrained brain capillaries, with BBB opening *in vitro* being primarily achieved due to the formation of standing waves generated by the wave reflection from the media-air interphase (as reported in 84). Although FUS^+MB^ has been previously shown to achieve BBB opening in such a configuration *in vitro* 18, 45, alternative model settings that create a more biologically and physically accurate environment for FUS^+MB^ exposure, might have higher translational capability. Finally, our model is currently limited to a fluorescence-based approach. Further improvements, such as incorporating for example high-performance liquid chromatography (HPLC) could allow for a wider range of drugs to be assessed.

In conclusion, our model provides an important advancement in the field of ultrasound-based therapies, as currently there are no patient cell-based platforms to screen for FUS^+MB^-deliverable drugs or investigate the effects of FUS^+MB^ at the cellular level in humans. Using a patient-derived cell platform enables more rapid translation of findings into the clinic due to the ability to capture patient heterogeneity, which is common amongst AD and other neurodegenerative disease patients. This platform can be used to screen for novel FUS-deliverable drugs that can then be tested in pre-clinical models and ultimately in patients. Finally, development of 2.5D and 3D BBB semi- or high-throughput models for FUS^+MB^ drug delivery screening will enable accelerated translation due to the ability to more closely mimic the human brain and brain cell interactions, downscale reagent use, and upscale the number of replicates. Importantly, since a 3D gel allows the aggregation of amyloid-β (Aβ) plaques *in vitro* 85, a multicellular 2.5/3D model system would enable the study of FUS^+MB^-mediated brain to blood Aβ clearance as well as modelling characteristics of the AD brain such as cerebral amyloid angiopathy. In addition, such a system would enable the interaction of FUS^+MB^-delivered drugs with AD pathologies, Aβ plaques and tau NFTs, to be studied, which is not possible in a 2D format. Our model also provides a potential platform to investigate nanoparticle-mediated drug delivery, with or without FUS^+MB^, which is a promising new avenue in the treatment of AD and other neurodegenerative diseases 86-89.

## Materials and methods

### Experimental design

The objective of this research study was to develop an *in vitro* BBB-like model for FUS^+MB^-mediated therapeutic antibody delivery and to investigate the contribution of the high-risk sporadic AD polymorphism, the *APOE4* allele, on FUS^+MB^ response. With such cell platforms not existing, we hypothesized that by using a patient-derived cell model, we could accelerate the translation of FUS^+MB^ treatment for therapeutic drug delivery into the clinic by identifying FUS^+MB^-deliverable drugs. In addition, since the effects of FUS^+MB^ on a cellular and molecular level are not known, our aim was to carefully investigate these using microscopy, viability and RNA-seq analysis and identify potential differences between low risk (*APOE3*) and high risk (*APOE4*) AD cells. This was a controlled laboratory experiment, utilizing iBEC generation from n = 3 *APOE3* carrier iPSC lines and n = 3 *APOE4* carrier iPSC lines, which included one isogenic pair (*APOE4* converted to *APOE3*). In addition, from the same lines as used for iBEC generation, n = 2 *APOE3* and *APOE4* lines, including one isogenic pair were used to generate iAstrocytes for co-culture experiments. Cells in mono- and co-cultures were exposed to FUS^+MB^ to investigate the effects of the treatment on cell molecular responses and to investigate FUS^+MB^-mediated therapeutic antibody delivery. Sample size and experimental replicates were selected based on previous experiments with the current iPSC lines as well as based on what is commonly accepted in the field based on literature. Experimental replicates and statistical tests used are specified in figure legends.

### Statistical analysis

Statistical analysis was performed using GraphPad Prism version 9.0.1. For normalized data (UT = 1), data were analyzed using a one-sample t-test comparing the values to mean = 1. For unnormalized values, data were analyzed using a two-tailed unpaired t-test with Welch's correction or with one-way ANOVA with multiple comparisons. A *P* value of less than < 0.05 was considered statistically significant. Values are shown as mean ± SEM or mean ± SD and specified in figure legends. The number of biological and independent replicates used for each experiment is specified in figure legends.

### Generation of human iPSC-derived brain endothelial-like cells for the *in vitro* BBB model and characterization of barrier integrity

Human iPSC lines were generated and characterized as previously described 37-39. iPSCs were expanded on human recombinant vitronectin in StemFlex^TM^ medium (Thermo Fisher Scientific). iBEC differentiation was performed as previously described by us 18. Briefly, iPSCs were first cultured for 6 days in an unconditioned medium, followed by 2 days in endothelial serum free medium (ESFM) supplemented with 2 % B-27, 10 μM and 20 ng/mL FGF-2, after which generated iBECs were purified on collagen IV and fibronectin coating 18. To establish a BBB *in vitro* model for antibody delivery experiments, iBECs were purified in Ø 0.4 μm or 3.0 Ø μm pore polyester or polycarbonate Transwell inserts (Sigma) in mono- or co-cultures with iAstrocytes (specified below). Forty-eight hours following iBEC purification 18, barrier integrity was characterized by measuring TEER using the EVOM3 Volt/Ohmmeter (World Precision Instruments). Passive dextran permeability was measured by culturing iBECs on Ø 0.4 μm pore polyester or polycarbonate Transwell inserts and cells were exposed to 0.5 mg/mL fluorescein isothiocyanate (FITC)-conjugated 3 - 5 kDa dextran (Sigma) for 24h. The top and bottom well fluorescence was measured (490 nm excitation/520 nm emission) using a plate reader (Biotek Synergy H4) and clearance volume calculated as previously described 40.

### Human iPSC-derived iAstrocyte culture and establishment of BBB co-cultures

For astrocyte differentiation, NPCs were first generated from iPSC lines using STEMdiff^TM^ SMADi Neural Induction kit (Stemcell technologies). Briefly, embryoid bodies (EBs) were first generated using the Aggrewell^TM^ plate for 5 days, after which EBs were plated on poly-L-ornithine and laminin coating for 7 days to generate neural rosettes. Rosettes were harvested and expanded in STEMdiff ^TM^ Neural Progenitor Medium. Generated NPCs were expanded until passage 5 and characterized for nestin and SOX2 expression to ensure adequate differentiation (Figure S5A). iAstrocyte differentiation of NPCs was initiated by switching the medium to astrocyte medium (DMEM/F12+GlutaMAX, 1 % N-2 supplement, 1 % fetal bovine serum, all from Thermo Fisher Scientific) as previously described 90 and continued for at least 60 days. Astrocyte maturation was initiated 7 days prior to experiments by exposing cells to 10 ng/mL BMP-4 and CNTF as previously described 56. BBB co-cultures were established by culturing iAstrocytes at the density of 5,000 cells/cm^2^ in astrocyte medium supplemented with BMP4 and CNTF for 7 days. iBECs seeded in collagen IV and fibronectin-coated Ø 3.0 μm Transwell inserts (Sigma) were placed in co-culture with astrocytes 24 h following purification and both cell types were maintained in human ESFM supplemented with 2% B-27 (Thermo Fisher Scientific). Co-cultures were maintained for 24 h prior to experiments.

### Focused ultrasound (FUS) and microbubble (MB) experiments

In this study, a FUS system with a center frequency of 286 kHz was used. The system consisted of a transducer (Sonic Concepts) having an active diameter of 64 mm, with a 63.2 mm radius curvature, housed in a 82 mm spherical shell with a central opening of Ø 20 mm used with the RF amplifier (Electronics & Innovation, Ltd). The focus of the transducer had approximate dimensions of 6.04 mm × 39.49 mm (Ø focal width x focal length). Transducer was mounted in a custom-made plexiglass holder and immersed in a water bath filled with de-gassed water. Transducer pressure wave calibration was performed using needle hydrophone. All experiments presented in this study were performed using the following FUS settings: 286 kHz center frequency, 0.3 MPa peak rarefactional pressure applied outside of the cell culture plate, 50 cycles/burst, burst period 20 ms, and a 120-s sonication time. Prior to FUS treatment, cells were exposed to phospholipid-shelled microbubbles with octafluoropropane gas core, prepared in-house, following previously described chemical synthesis protocol 91. The generated MBs were characterized for their concentration and size using a Multisizer 4e coulter counter (Beckman Coulter) as previously described 20. The average concentration of MBs used in our study was 7.47 ± 6.06 x 10^9^/ml with a diameter of 1.24 ± 0.31 µm (Figure S9). For dextran and antibody delivery studies, iBECs were seeded on Ø 3.0 μm pore Transwells at 1.4x10^6^ cells/cm^2^. The effect of FUS^+MB^ was tested on iBECs 48-72h after subculture on Transwell inserts. FITC-conjugated dextran (150 kDa) was added at 0.5 mg/ml and AlexaFluor™-647-conjugated anti-amyloid-β (Aducanumab-analogue, 27) and anti-Tau (RNF5, 36) therapeutic antibodies were added at 1 μM. MBs (10 μL per Transwell) were then added to the wells aseptically directly before the FUS treatment. Cells were then exposed to FUS and 24 h after FUS treatment, media samples from top and bottom chambers of the Transwell were collected for spectrofluorometric analysis. Fluorescence of dextran was measured at 490 nm excitation/520 nm emission and fluorescence of antibodies was measured at 633 nm excitation/665 nm emission using a plate reader (Biotek Synergy H4). Clearance volume in UT and FUS^+MB^ treated Transwells was calculated as previously described 40 and data presented as fold change relative to UT. For the assessment of FUS^only^ and FUS^+MB^ on iBEC monolayer integrity, iBECs cultured on Ø 3.0 μm pore Transwells were exposed to FUS^only^ or FUS^+MB^ and TEER measured 1 h post treatment. Cell culture media was then replaced with fresh ESFM + B-27, cells placed back in the incubator and TEER measured again at 24 h. Fold change in TEER compared to UT controls was then calculated. For the assessment of FUS^only^ and FUS^+MB^ on iBEC permeability, iBECs cultured on Ø 0.4 μm pore Transwells were exposed to FUS^only^ or FUS^+MB^ and FITC-conjugated dextran (5 kDa; 0.5 mg/ml, incubated for 1 h) permeability was measured 1 h post treatment as described above. Subsequently, cell culture media was replaced with fresh ESFM + B27, cells placed back in the incubator 5 kDa dextran re-applied 24 h later and permeability measured. Fluorescence intensity of dextran in the media collected from the bottom chamber of the Transwell was measured as described above and data presented as fold change relative to UT.

### Lactate dehydrogenase (LDH) cytotoxicity assay

To assess the effects of FUS^+MB^ on iBEC and iAstrocyte viability, cell culture media samples were collected 1 h and 24 h after FUS^+MB^ exposure and stored at -80 °C until analysis. The level of lactate dehydrogenase (LDH) enzyme in the collected media was determined using CyQUANT LDH Cytotoxicity Assay (Thermo Fisher Scientific) following the manufacturer's instructions. Absorbance was measured at 490 nm and 680 nm using a plate reader. To determine LDH activity, the 680 nm absorbance value (background) was subtracted from the 490 nm absorbance and compared between treatment conditions.

### RNA extraction, cDNA synthesis and quantitative real-time PCR

For RNA collection, cells were rinsed with PBS, exposed to TRIzol^TM^ reagent and scraped off the culture plate using a pipette tip. Total RNA was extracted using the Direct-zol RNA Miniprep Kit (Zymo Research) according to the manufacturer's instructions and treated in-column with DNase I. RNA quality and quantity was measured using NanoDrop^TM^ Spectrophotometer, after which RNA was converted to cDNA using SensiFAST^TM^ cDNA synthesis kit (Bioline) according to the manufacturer's instructions. The qPCR run was performed as triplicate for each sample on QuantStudio^TM^ 5 Real-Time PCR system with run conditions as follows: 2 min at 95 °C followed by 40 cycles of 5 s at 95 °C and 30 s at 60 °C. Ct values were normalized to Ct values of 18S endogenous control (ΔCt values), which were found to be consistent across cell lines, conditions and timepoints. ΔΔCt values were calculated as 2^(-ΔCt)^ and presented as ΔΔCt multiplied by 10^6^ or as fold change. Primer sequences used in this study are presented in Table S3.

### Bulk RNA-sequencing (RNA-seq)

For transcriptome analysis of FUS^+MB^ treated iBECs, bulk RNA-seq was performed. Briefly, iBECs were purified on collagen IV- and fibronectin-coated 24-well plates, then treated with 20 μL of MBs followed by FUS with parameters described above. RNA samples were harvested at 1 h and 24 h timepoints from untreated and FUS^+MB^ treated cells. Total RNA was extracted as described above. The quality of total RNA was determined using the Agilent TapeStation system. In total, 24 samples underwent RNA-seq: samples from 6 patients (n = 3 *APOE3* and n = 3 *APOE4*) each with FUS^+MB^ treatment or UT at both 1 h and 24 h timepoints.

Library preparation was performed using Illumina TruSeq Stranded mRNA kit and libraries were sequenced using the Illumina NextSeq 550 platform at the QIMR Berghofer Next Generation Sequencing facility with 75 bp reads sequenced to ~ 40 million reads per sample. Sequence reads were trimmed for adaptor sequences using Cutadapt version 1.9 92 and aligned using STAR version 2.5.2a 93 to the human GRCh37 assembly with the gene, transcript, and exon features of Ensembl (release 70) gene model. Quality control metrics were computed using RNA-SeQC version 1.1.8 94 and expression was estimated using RSEM version 1.2.30 95.

All downstream RNA-seq analysis was performed using R version 3.6.2. Differential expression analysis was performed using edgeR's quasi-likelihood pipeline version 3.28.0 96-98. Specifically, only protein-coding genes that passed the minimum expression filter using edgeR's filterByExpr function with default settings were kept for further analysis. Two design matrices were constructed for the analyses herein. For the comparisons between FUS^+MB^ treatment vs UT at different time points irrespective of genotype, an additive linear model was used, which incorporated a patient term and the remaining experimental conditions combined into one factor. Specifically, we used model.matrix(~Patient + Treatment.Time), where Patient was one of 6 patient IDs, Treatment is UT or FUS^+MB^, and Time is 1 h or 24 h. For the comparisons between genotypes, all experimental conditions were combined into a single factor. Specifically, we used model.matrix(~Genotype.Treatment.Time), where Genotype is *APOE3* or *APOE4*, and Treatment and Time as per above. The glmQLFit() function was used to fit a quasi-likelihood negative binomial generalized log-linear model to the read counts for each gene. Using the glmQLFTest() function, we conducted gene-wise empirical Bayes quasi-likelihood F-tests for a given contrast. Differentially expressed genes (DEGs) were determined using a false discovery rate (FDR) < 0.05. To perform gene ontology (GO) term analysis, multiple functions from the clusterProfiler package version 3.14.3 were utilized. First, the bitr function was used to convert gene IDs of DEGs from Ensembl to Entrez. Entrez IDs were subsequently passed to the enrichGO function, before plotting the results with the dotplot function 99.

### Immunofluorescence

For immunofluorescence characterization, iBECs grown on collagen IV and fibronectin, washed with PBS and fixed with ice-cold 100 % methanol for 5 min or 4 % paraformaldehyde (PFA) for 15 min. Astrocytes were fixed with 4 % PFA. Following PFA fixing, cells were permeabilized for 10 min with 0.3 % Triton-X. Cells were then blocked for 1 - 2 h at RT with 2 % bovine serum albumin and 2 % normal goat serum in PBS. Primary antibodies (Table S4) were diluted in a blocking solution and incubated overnight at 4 °C. The following day, cells were washed three times with PBS, then incubated with secondary antibodies (Table S4) diluted in blocking solution for 1 - 2 h at RT in the dark. Finally, cells were washed three times with PBS, Hoechst counterstaining was performed, and coverslips were mounted with ProLong Gold Antifade (Thermo Fisher Scientific). Images were obtained at 10X or 20X magnification using a Zeiss 780 confocal microscope. Image brightness was increased for presentation purposes using ImageJ.

### 2.5D BBB-like model

The 2.5D BBB-like model was established using photo-crosslinkable synthetic LunaGel^TM^ (Gelomics, Brisbane, Australia). For this, iAstrocytes were first seeded in the gel prior to iBEC seeding. Briefly, iAstrocytes were differentiated for approximately 60 days, after which they were detached from their culture vessel with TrypLE (Thermo Fisher Scientific) and a cell count was performed. iAstrocytes were then centrifuged to a pellet (300 x g, 5 min) and resuspended in LunaGel^TM^ mixed 1:1 with photoinitiator as per the manufacturer's instructions. iAstrocytes in the gel were then seeded in a clear 96-well plate at a ratio of 10,000 cells per 50 μL of gel per well. The gel was polymerized for 30 s using the LunaCrosslinker^TM^ to ensure a soft gel for iAstrocyte proliferation. Following polymerization, 100 μL of astrocyte medium was added on top of the gel. The following day cells were supplemented with BMP-4 and CNTF for 7 days after which seeding of iBECs was performed. Prior to seeding iBECs on the iAstrocyte containing LunaGel^TM^, iBECs were purified on a collagen IV and fibronectin-coated culture flask for 24 h. Before iBEC seeding, astrocyte medium was removed, and a thin layer (25 μL) of high stiffness LunaGel^TM^ was seeded on top of the iAstrocyte layer and polymerized for 60 s. The layer of high stiffness gel was then coated with collagen IV and fibronectin for 1 h prior to seeding of iBECs. iBECs were detached using TrypLE and a cell count was performed. iBECs were resuspended in ESFM + B27 supplemented with 10 μM retinoic acid, 10 μM ROCKinhibitor and 1.3 μM of hydrocortisone as previously described 100, 101. The coating solution was removed from the gel and iBECs seeded at 150,000 cells per well in 150 μL of supplemented ESFM+B27 on top of the iAstrocyte gel. iBECs were allowed to attach for 24 h, after which the medium was switched to ESFM+B27 with no supplementation. For assessment of barrier formation on the LunaGel^TM^, iBECs were seeded on layers of soft and stiff gel in supplemented ESFM+B27 as described above and allowed to attach for 24 h. Culture medium was then changed to ESFM+B27 without supplements and cells rested for 2 h. Dextran (5 kDa; 0.5 mg/ml) was applied and incubated for 2 h. Dextran was then removed, replaced with PBS and gel fluorescence intensity was measured as described above using a plate reader. Aducanumab delivery using FUS^+MB^ was performed as described above. After 24 h, the supernatant was removed and replaced with PBS. Antibody fluorescence intensity within the gel was measured using a plate reader as described above. Immunofluorescent staining of LunaGel^TM^ cultures was performed as described above with extended incubation times for 4% PFA fixing (1 h) and permeabilization (30 min). Imaging was performed using a Zeiss 780 confocal microscope (iAstrocytes) or ANDOR WD Revolution Spinning Disk microscope (iBECs).

## Supplementary Material

Supplementary figures and tables S3-S4.

Supplementary tables S1.

Supplementary tables S2.

## Abbreviations

AJ: adherens junction
APOE: apolipoprotein E
AQP4: aquaporin-4
Aβ: amyloid-β
BBB: blood-brain barrier
BEC: brain endothelial cell
BMP-4: bone morphogenic protein 4
CCL2: C-C motif chemokine ligand 2
CNTF: ciliary neurotrophic factor
DEG: differentially expressed gene
ESFM: endothelial serum-free medium
FDR: false discovery rate
FUS: focused ultrasound
GFAP: glial fibrillary acidic protein
hiPSC: human induced pluripotent stem cell
HPLC: high-performance liquid chromatography
iBEC: induced brain endothelial-like cell
IL: interleukin
LDH: lactate dehydrogenase
MB: microbubble
NFT: neurofibrillary tangle
NPC: neural progenitor cell
p-Tau: phosphorylated Tau
PCA: principal component analysis
RNA-seq: RNA sequencing
TEER: trans-endothelial electrical resistance
TJ: tight junction
UT: untreated
