# Supplementary material for: A sporadic Alzheimer's blood-brain barrier model for developing ultrasound-mediated delivery of Aducanumab and anti-Tau antibodies
Source: Theranostics. 2022 Sep 25;12(16):6826–47. doi: 10.7150/thno.72685 (PMC9576609; doi:10.7150/thno.72685)
Supplement: Supplementary file 1 — Supplementary figures and tables S3-S4. [file thnov12p6826s1.pdf]

SUPPLEMENTARY MATERIAL

A sporadic Alzheimer's blood-brain barrier model for developing ultrasound-mediated delivery of Aducanumab and anti-Tau antibodies

Wasielewska *et al*.

SUPPLEMENTAL FIGURES

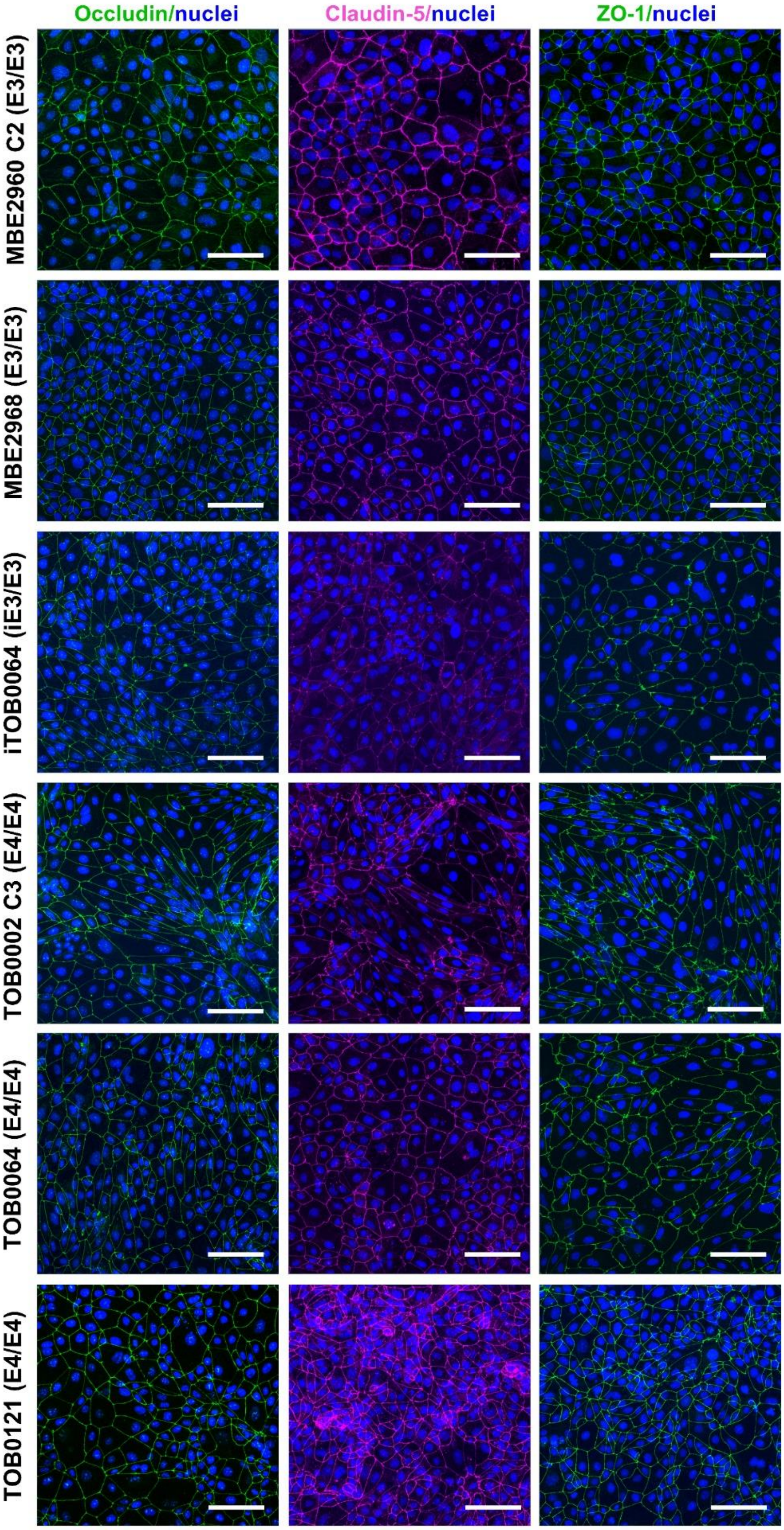

Figure S1. Characterization of iBECs generated from individual iPSC lines. Immunofluorescence images of occludin (green), claudin-5 (magenta) and ZO-1 (green) in

iBECs generated from *APOE3* and *APOE4* induced pluripotent stem cells (iPSCs) used in this study. Hoechst counterstain, scale bar = 100 μm.

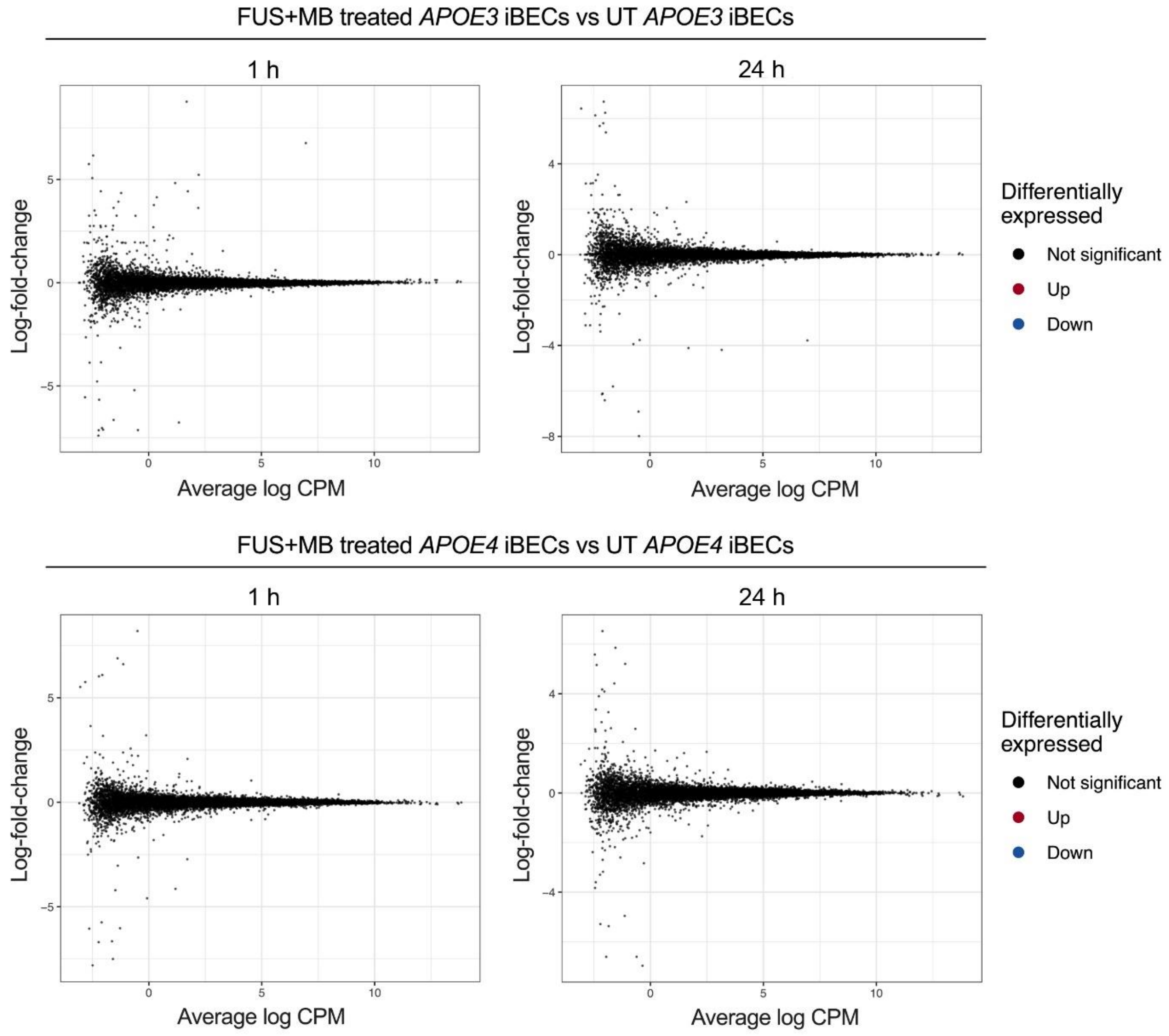

Figure S2. Differential expression analysis of FUS^{+MB} treated iBEC vs untreated iBECs at 1 h and 24 h for *APOE3* and *APOE4* genotypes. Mean-difference (MD) plots showing log-fold-change versus average log expression values (log2 counts per million, CPM). Upper panel: FUS^{+MB} treated *APOE3* iBECs at 1 h vs untreated (UT) *APOE3* iBECs at 1 h and FUS^{+MB} treated *APOE3* iBECs at 24 h vs UT *APOE3* iBECs at 24 h. Lower panel: FUS^{+MB} treated *APOE4* iBECs at 1 h vs UT *APOE4* iBECs at 1 h and FUS^{+MB} treated *APOE4* iBECs at 24 h vs UT *APOE4* iBECs at 24 h.

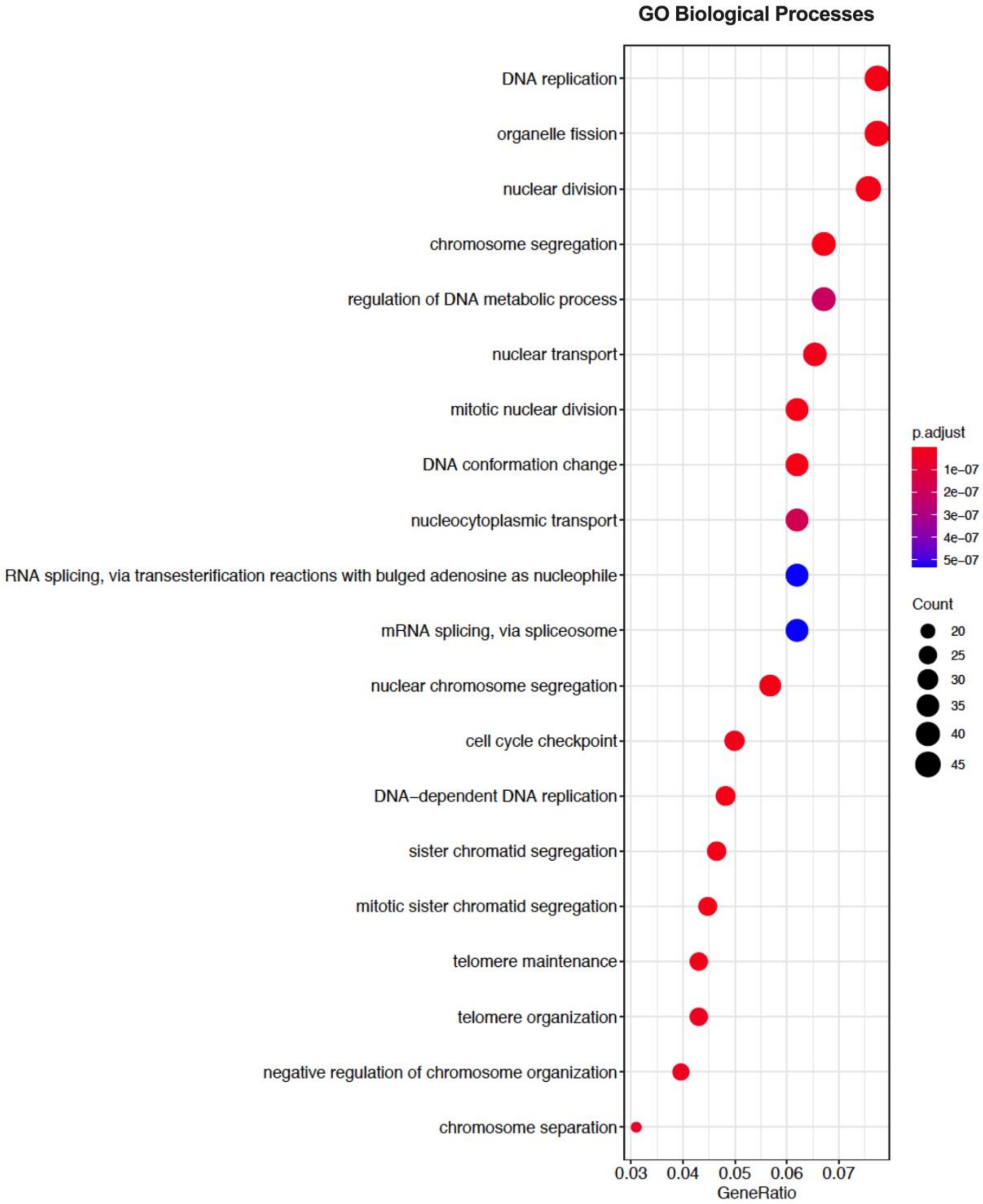

Figure S3. Dotplot of top gene ontology (GO) terms from sub ontology Biological Process enriched from comparison of UT *APOE4* iBECs at 1 h vs *APOE3* iBECs at 1 h. The top 20 GO processes according to *P*-value plotted in order of gene ratio. The size of the dots represents the number of genes associated with the GO term and the color of the dots represent the *P*-adjusted values. The differentially expressed genes (FDR < 0.05) were used for analysis.

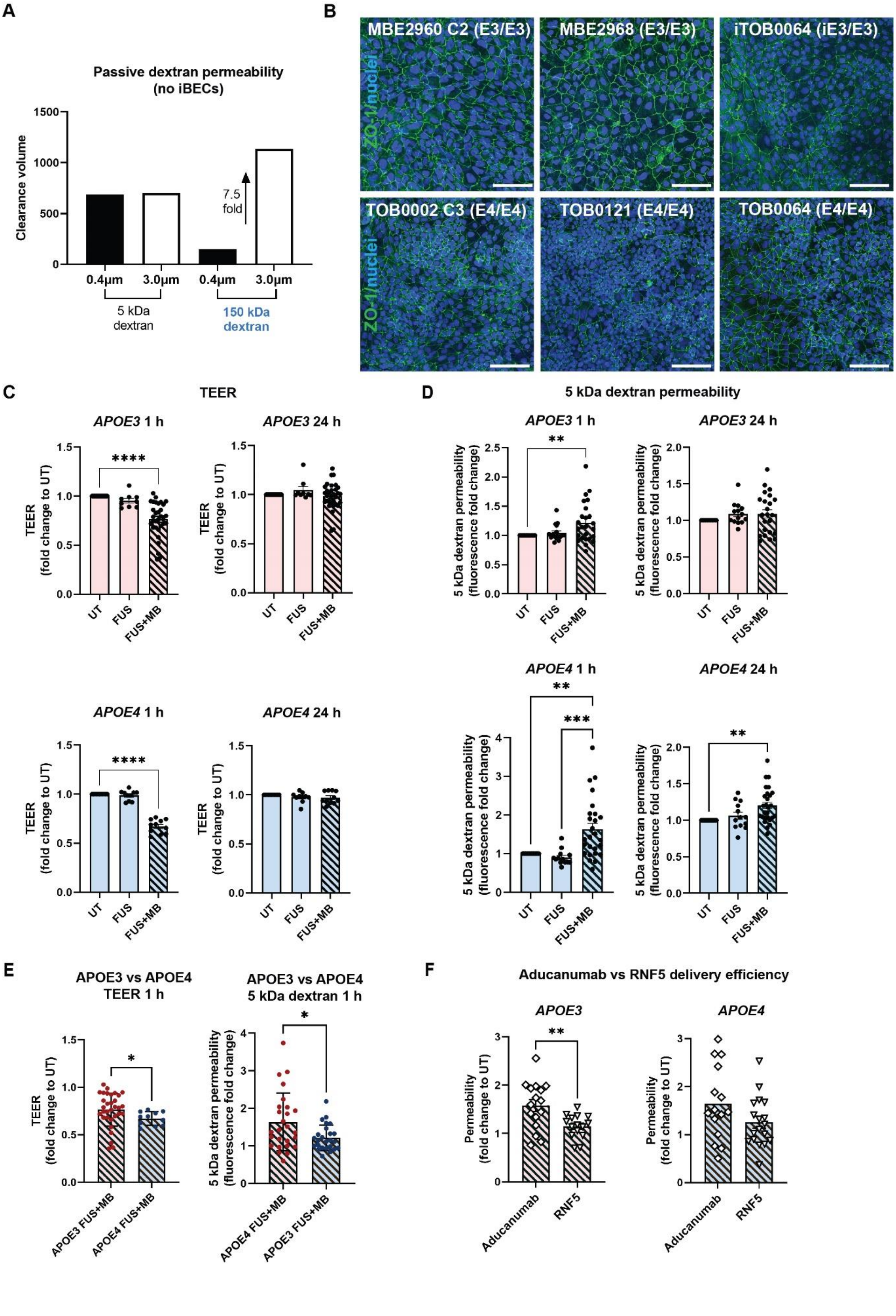
A
Passive dextran permeability
(no iBECs)
Clearance volume
1500
1000
500
0
7.5
fold
0.4µm
3.0µm
0.4µm
3.0µm
5 kDa
dextran
150 kDa
dextran
B
MBE2960 C2 (E3/E3)
MBE2968 (E3/E3)
iTOB0064 (iE3/E3)
TOB0002 C3 (E4/E4)
TOB0121 (E4/E4)
TOB0064 (E4/E4)
ZO-1/nuclei
ZO-1/nuclei
C
TEER
APOE3 1 h
APOE3 24 h
APOE4 1 h
APOE4 24 h
TEER
(fold change to UT)
****
****
UT
FUS
FUS+MB
D
5 kDa dextran permeability
APOE3 1 h
APOE3 24 h
APOE4 1 h
APOE4 24 h
5 kDa dextran permeability
(fluorescence fold change)
**
**
***
**
UT
FUS
FUS+MB
E
APOE3 vs APOE4
TEER 1 h
APOE3 vs APOE4
5 kDa dextran 1 h
*
*
TEER
(fold change to UT)
5 kDa dextran permeability
(fluorescence fold change)
APOE3 FUS+MB
APOE4 FUS+MB
APOE4 FUS+MB
APOE3 FUS+MB
F
Aducanumab vs RNF5 delivery efficiency
APOE3
APOE4
**
Permeability
(fold change to UT)
Aducanumab
RNF5

Figure S4. Optimization of the Ø 3.0 µm pore Transwell model and characterization of the effects of FUS^{only} and FUS^{+MB} on iBECs. (**A**) Passive permeability (clearance volume) of 5 kDa and 150 kDa dextran in collagen IV and fibronectin coated (no iBEC containing) Ø 0.4 µm and Ø 3.0 µm pore Transwell inserts. (**B**) Immunofluorescence of ZO-1 (green) in each individual *APOE3* and *APOE4* iBEC line seeded on Ø 3.0 µm pore Transwell inserts (Hoechst counterstain, scale bar = 100 µm). (**C**) Trans-endothelial electrical resistance (TEER, fold change to untreated (UT)) in *APOE3* and *APOE4* iBECs in UT, FUS^{only} and FUS^{+MB} conditions at 1 h and 24 h following treatment (N = 2 biological replicates and a minimum of n = 3 independent replicates per line). (**D**) 5 kDa dextran permeability (fluorescence fold change to UT) in *APOE3* and *APOE4* iBECs in UT, FUS^{only} and FUS^{+MB} conditions at 1 h and 24 h following treatment (N = 2 biological replicates and a minimum of n = 3 independent replicates per line). (**E**) Comparison of TEER and 5 kDa dextran permeability following FUS^{+MB} between *APOE3* and *APOE4* iBECs (permeability shown as relative values to UT at 1 h). (**F**) Comparison of Aducanumab-analogue and RNF5 delivery efficiency following FUS^{+MB} in *APOE3* and *APOE4* iBECs (permeability shown as relative values to UT at 24 h). Error bars = SEM. * $P < 0.05$, ** $P < 0.01$, *** $P < 0.001$ and **** $P < 0.0001$ by one-way ANOVA for graphs with three groups and by Unpaired t-test with Welch's correction for graphs with two groups.

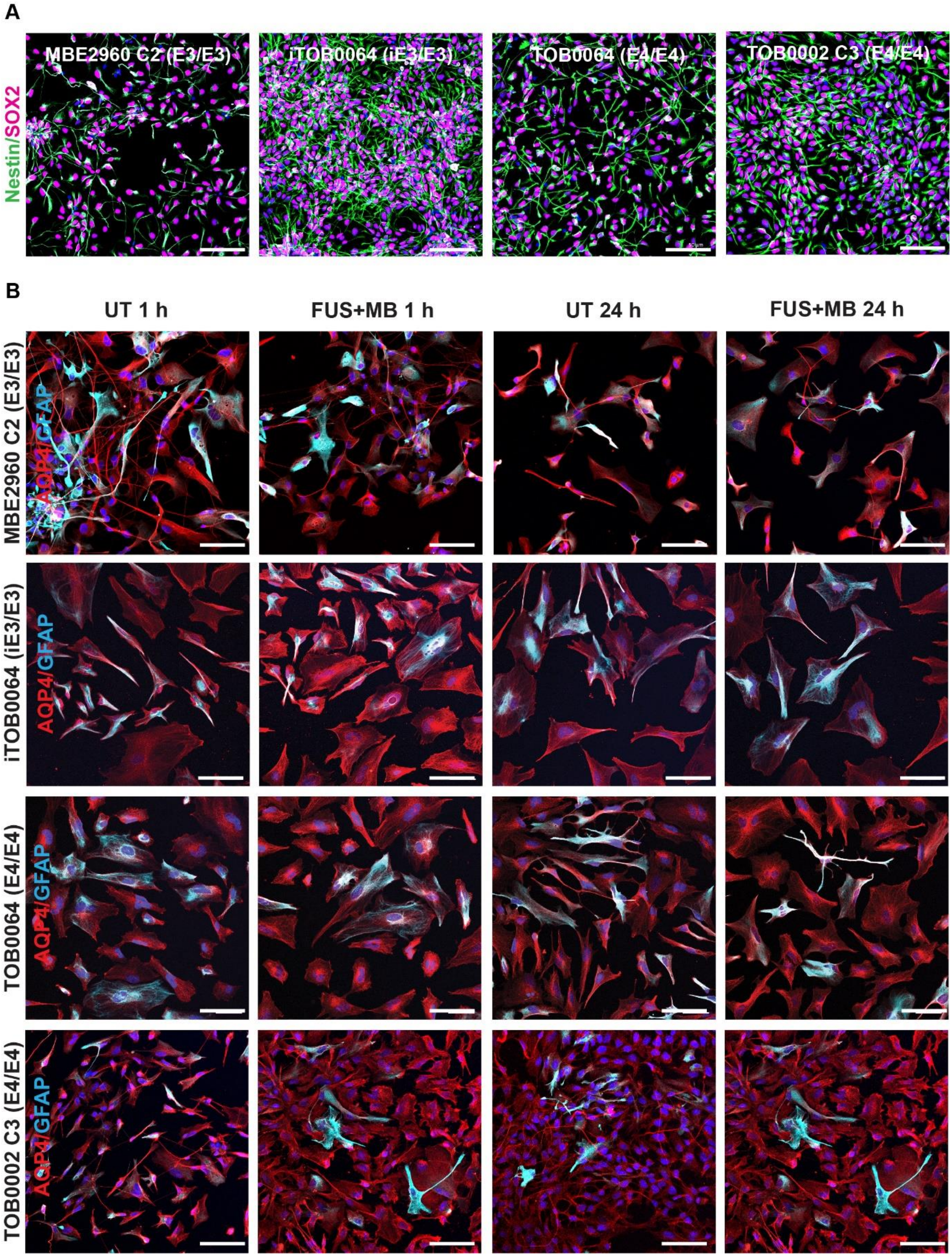

Figure S5. **Neural progenitor cell generation and FUS^{+MB} treatment of iAstrocytes.** (**A**) Immunofluorescence of nestin (green) and SOX2 (magenta) in neural progenitor cells (NPCs) generated from *APOE3* and *APOE4* induced pluripotent stem cells (iPSCs) used in this study. (**B**) Immunofluorescence of *APOE3* and *APOE4* iAstrocytes generated from individual iPSC

lines in untreated (UT) and focused ultrasound + microbubble (FUS^{+MB}) conditions 1 h and 24 h following treatment stained with AQP4 (red) and GFAP (cyan) (Hoechst counterstain, scale bar = 100 μm).

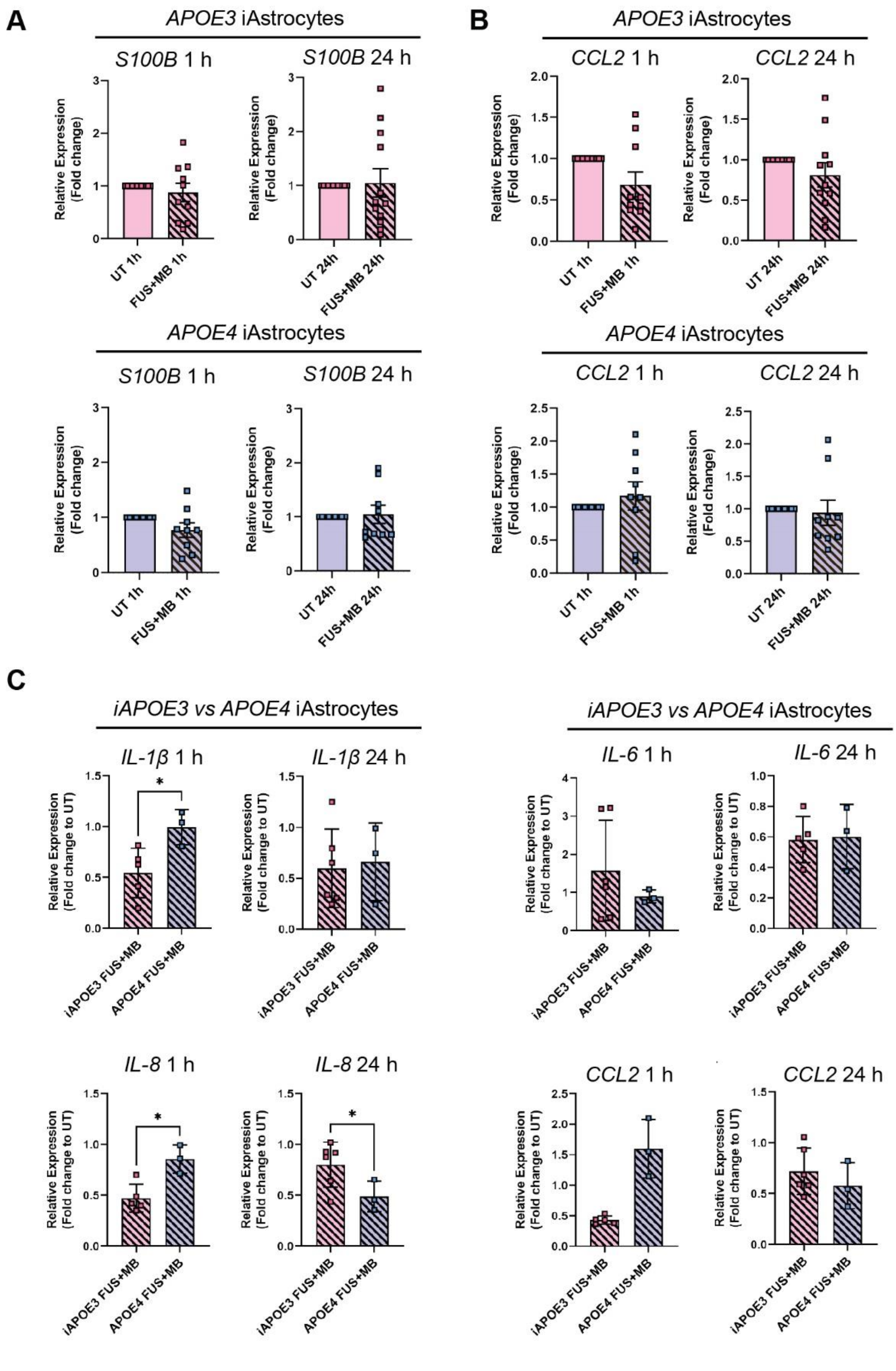

Figure S6. **Effect of FUS^{+MB} on iAstrocyte gene expression.** Relative gene expression (fold change) of (**A**) astrocyte marker *S100B* and (**B**) inflammatory cytokine *CCL2* in UT and FUS^{+MB} treated *APOE3* and *APOE4* iAstrocytes 1 h and 24 h after treatment, error bars = SEM. (**C**) Comparison of relative gene expression (fold change) of inflammatory markers between

an isogenic *iAPOE3* and *APOE4* iAstrocyte pair at 1 h and 24 h after FUS^{+MB} treatment, error bars = SD. * $P < 0.05$ by Unpaired t-test with Welch's correction.

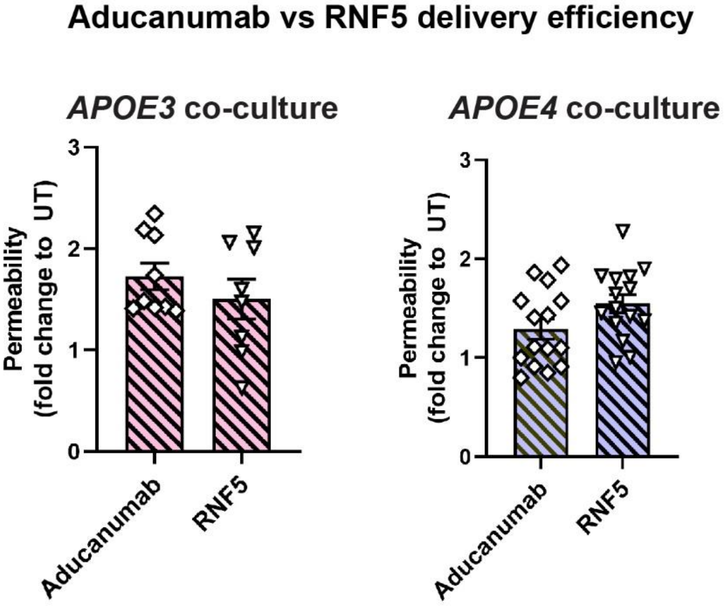

Figure S7. Comparison of Aducanumab-analogue and RNF5 delivery efficiency following FUS^{+MB} in *APOE3* and *APOE4* co-cultures. Aducanumab and RNF5 delivery efficiency following FUS^{+MB} in *APOE3* and *APOE4* iBEC and iAstrocyte co-cultures (permeability shown as relative values to UT at 24 h).

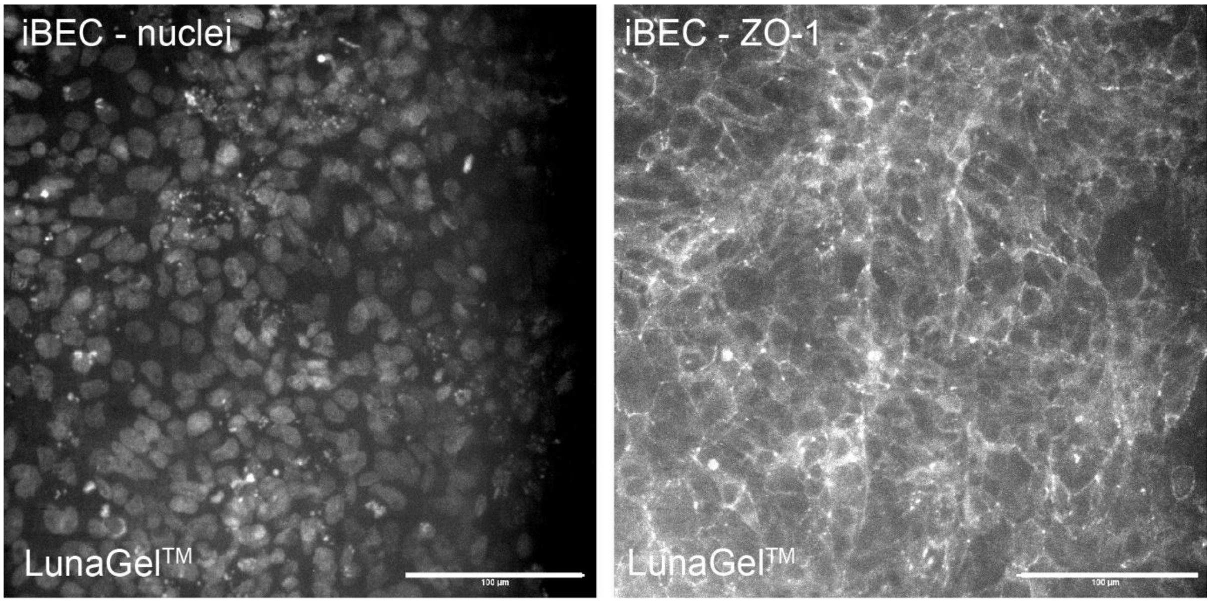

Figure S8. iBEC barrier formation on LunaGel™. Greyscale images of Hoechst staining (left panel) and ZO-1 staining (right panel) in iBECs seeded on LunaGel™ (scale bar = 100 μm).

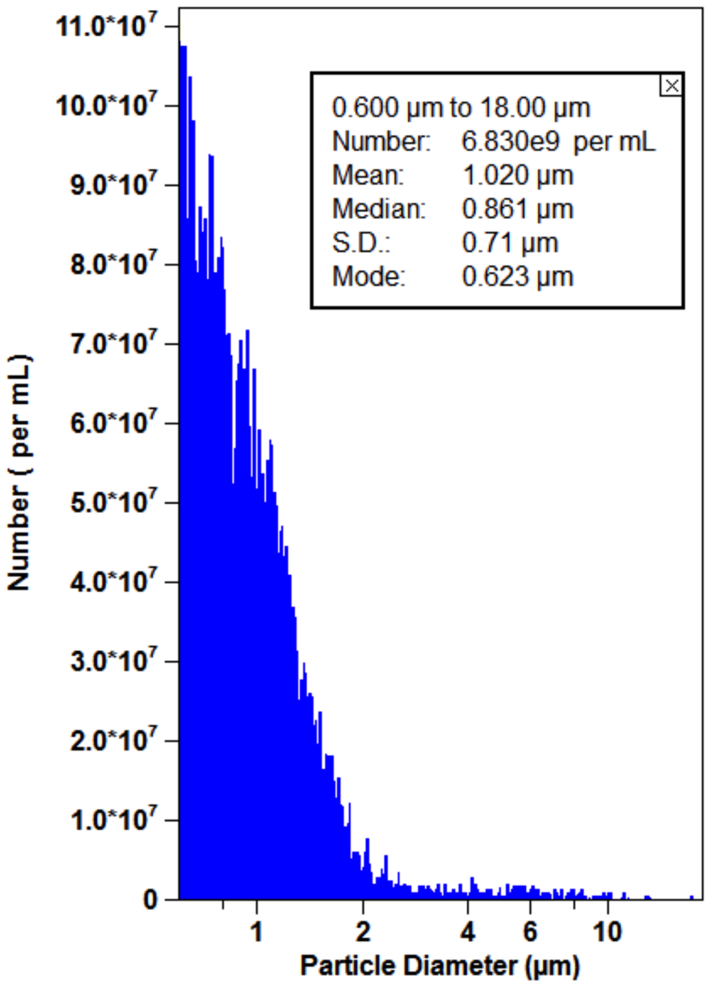

Figure S9. Characterization of microbubbles used in the study. Representative size and distribution profiles of in-house prepared gas-filled microbubbles measured with a Coulter counter. The average diameter of microbubbles used in the study was 1.24 ± 0.31 µm, with a concentration of 7.47 ± 6.06 × 10^9 MBs/ml (n = 6 MB vials analysed).

SUPPLEMENTAL TABLES

Table S1. Results from differential expression analysis of UT *APOE4* iBECs compared to UT *APOE3* iBECs at 1 h. Genes sorted by *P*-value. Differentially expressed genes (DEGs) defined by FDR < 0.05.

Table can be found in .xlsx format

Table S2. Results from gene ontology (GO) enrichment analysis of sub ontology Biological Process from comparison of UT *APOE4* iBECs at 1 h vs *APOE3* iBECs at 1 h.

Table can be found in .xlsx format

Table S3. Primer sequences used in the study.

**Target gene**	**Forward primer sequence**	**Reverse primer sequence**
Ve-cadherin *(CDH5)*	AGGCAAGATCAAGTCAAGCGT	GAGTCTCCAGGTTTTCGCCA
Claudin-5 *(CLDN5)*	GATTGAGAGGTCTGGGAAGCC	ATCCCATGGCAAACAGAGAGG
Occludin *(OCLN)*	GAAGCAAGTGAAGGGATCTGC	ACAACTTGGCATCAGCCTTCT
Zonula occludens-1 *(TJP-1)*	ACAGCTACAGGAAAATGACCGA	ACTGGTTCAGGATCAGGACG
SOX18	TCAGCAAGATGCTGGGCAAAG	GCGGCCGGTACTTGTAGTTG
Interleukin-6 *(IL-6)*	TGCAATAACCACCCCTGACC	TGCGCAGAATGAGATGAGTTG
Interleukin-8 *(IL-8)*	AGACAGCAGAGCACACAAGC	ATGGTTCCTTCCGGTGGT
Interleukin-1β *(IL-β)*	AATCTGTACCTGTCCTGCGTGTT	TGGGTAATTTTTGGGATCTACACTCT
C-C motif chemokine ligand 2 *(CCL2)*	GCTCATAGCAGCCACCTTCATTC	GGACACTTGCTGCTGGTGATTC
Glial fibrillary acidic protein *(GFAP)*	GAGGTTGAGAGGGACAATCTGG	GTGGCTTCATCTGCTTCCTGTC
Aquaporin-4 *(AQP-4)*	GTAGTCACCATGGTTCATGGAAAT	TGGAACACAGCTGGCAAAGA
S100 calcium-binding protein β *(S100β)*	TTCTGGAAGGGAGGGAGACA	CTCCTGCTCTTTGATTTCCTCT
18S	TTCGAGGCCCTGTAATTGGA	GCAGCAACTTTAATATACGCTATTGG

Table S4. Antibodies used in the study.

**Primary antibodies**	**Species**	**Source**	**Identifier**
ZO-1	mouse	Invitrogen	Cat# 339100
occludin	rabbit	Invitrogen	Cat# 711500
claudin-5	mouse	Invitrogen	Cat# 352500
aquaporin-4	mouse	Abcam	Cat# ab9512
GFAP	rabbit	Agilent/ Dako	Cat# Z0334
nestin	mouse	Abcam	Cat# ab22035
SOX2	rat	Invitrogen	Cat# 14981182
**Secondary antibodies**	**Species**	**Source**	**Identifier**
anti-mouse Alexa Fluor 488	goat	Invitrogen	Cat# A11029
anti-mouse Alexa Fluor 594	goat	Invitrogen	Cat# A11032
anti-mouse Alexa Fluor 647	goat	Invitrogen	Cat# A32728
anti-rabbit Alexa Fluor 488	goat	Invitrogen	Cat# A11034
anti-rat Alexa Fluor 647	goat	Invitrogen	Cat# A21247
